# Health of singleton neonates in Switzerland through time and crises: a cross-sectional study at the population level, 2007-2022

**DOI:** 10.1186/s12884-024-06414-1

**Published:** 2024-03-25

**Authors:** Mathilde Le Vu, Katarina L. Matthes, Marek Brabec, Julien Riou, Veronika W. Skrivankova, Irene Hösli, Sabine Rohrmann, Kaspar Staub

**Affiliations:** 1 Institute of Evolutionary Medicine, University of Zurich, Zurich, Switzerland; 2 Institute of Computer Science of the Czech Academy of Sciences, Prague, Czech Republic; 3 Center for Primary Care and Public Health (Unisanté), University of Lausanne, Lausanne, Switzerland; 4 Institute of Social and Preventive Medicine (ISPM), University of Bern, Bern, Switzerland; 5 Department of Obstetrics and Gynaecology, University Hospital of Basel, Basel, Switzerland; 6 Epidemiology, Biostatistics, and Prevention Institute, University of Zurich, Zurich, Switzerland; 7 Swiss School of Public Health (SSPH+), Zurich, Switzerland

**Keywords:** Birth weight, Neonatal health, Crises, COVID-19, Pandemics

## Abstract

**Background:**

Being exposed to crises during pregnancy can affect maternal health through stress exposure, which can in return impact neonatal health. We investigated temporal trends in neonatal outcomes in Switzerland between 2007 and 2022 and their variations depending on exposure to the economic crisis of 2008, the flu pandemic of 2009, heatwaves (2015 and 2018) and the COVID-19 pandemic.

**Methods:**

Using individual cross-sectional data encompassing all births occurring in Switzerland at the monthly level (2007-2022), we analysed changes in birth weight and in the rates of preterm birth (PTB) and stillbirth through time with generalized additive models. We assessed whether the intensity or length of crisis exposure was associated with variations in these outcomes. Furthermore, we explored effects of exposure depending on trimesters of pregnancy.

**Results:**

Over 1.2 million singleton births were included in our analyses. While birth weight and the rate of stillbirth have remained stable since 2007, the rate of PTB has declined by one percentage point. Exposure to the crises led to different results, but effect sizes were overall small. Exposure to COVID-19, irrespective of the pregnancy trimester, was associated with a higher birth weight (+12 grams [95% confidence interval (CI) 5.5 to 17.9 grams]). Being exposed to COVID-19 during the last trimester was associated with an increased risk of stillbirth (odds ratio 1.24 [95%CI 1.02 to 1.50]). Exposure to the 2008 economic crisis during pregnancy was not associated with any changes in neonatal health outcomes, while heatwave effect was difficult to interpret.

**Conclusion:**

Overall, maternal and neonatal health demonstrated resilience to the economic crisis and to the COVID-19 pandemic in a high-income country like Switzerland. However, the effect of exposure to the COVID-19 pandemic is dual, and the negative impact of maternal infection on pregnancy is well-documented. Stress exposure and economic constraint may also have had adverse effects among the most vulnerable subgroups of Switzerland. To investigate better the impact of heatwave exposure on neonatal health, weekly or daily-level data is needed, instead of monthly-level data.

**Supplementary Information:**

The online version contains supplementary material available at 10.1186/s12884-024-06414-1.

## Introduction

Neonatal health is a major public health concern: it reflects the overall health and well-being of a population [1, 2]. It is therefore crucial to understand how it changes over time and which factors influence it the most. Neonatal health in a population can be assessed by indicators such as the proportion of stillbirth, preterm birth (PTB) or neonatal mortality, or by the average size and weight of newborns. These parameters can then be used to predict postnatal health and even long-term health outcomes, such as mortality and the likelihood of developing various health problems, including hypertension or cardiovascular diseases [3, 4].

Birth weight is extensively used as a proxy to estimate neonatal health and population health in general [5]. It is known to influence infant mortality and morbidity [6, 7], and can vary depending on genetics and/or lifestyle of the mother. Maternal infections occurring during pregnancy can also affect birth weight as well as risks of PTB and stillbirth [8–10]. PTB is a major determinant of neonatal health: it is the leading cause of neonatal mortality in otherwise healthy neonates, and premature infants are at a higher risk of developing diseases [11]. Risks of spontaneous PTB include a previous PTB [12], older age [13] or ethnicity [14], and are more common for multiple and/or technology-assisted pregnancies [12]. A third of all PTB are iatrogenic: these are elective due to maternal or foetal complications [15]. PTB is also associated with long-term negative outcomes such as cardiac, respiratory and neurodevelopmental impacts [16].

There are reports of minor birth weight increases in the second half of the 20^th^ century [17], but more recent variations are country-dependent [17–21]. In Europe, recent PTB time trends are also heterogeneous [22–24], but globally, PTB rates barely changed between 2010 and 2020 [16, 25]. High income countries (HICs) are also concerned, with the US being the 6^th^ country with the highest PTB rate worldwide [25]. Stillbirth rates in Western Europe and North America dramatically declined from the late 1930s, thanks to improved antenatal and intrapartum care and to the introduction of antibiotics [26]. In 2019, stillbirth rate was 2.9/1000 births in Western Europe: this is 26% lower than in 2000 [27], and this pattern is similar among HICs [27, 28]. However, this declining stillbirth rate has been slowing down recently [29].

When pregnant women are exposed to crises such as wars, pandemics or economic depressions, adverse neonatal health outcomes can occur more frequently, due to stress [30]. For instance, exposure to heatwaves reportedly makes pregnancies more vulnerable to PTB and stillbirth [31–33], while their impact on birth weight is still unclear [34]. Economic uncertainty may also indirectly influence neonatal health: during the economic crisis of 2008 (thereafter called the Great Recession) and its aftermaths, pregnancy outcomes worsened [35, 36], but aggregated-level studies fail to reach consistent results [37]. The Dutch famine of 1944-45 had immediate consequences on birth weight [38] and long-lasting consequences on the offspring of mothers who were exposed to it during pregnancy [39]. During the 1918 flu pandemic, higher rates of low birth weight (LBW *i.e.* birth weight <2’500g) [40], stillbirth and neonatal mortality were also reported [41]. Interestingly, birth cohorts exposed to this pandemic *in utero* had a shorter height and a lower socioeconomic status later on [42, 43]. The impact of the Coronavirus disease 2019 (COVID-19) pandemic on pregnancies is still unclear, but studies suggest that the mental health of pregnant women has worsened [44, 45], with up to 12% of pregnant women in Switzerland presenting depression- and anxiety-like symptoms during the pandemic [46]. Regarding effects on neonatal health, a meta-analysis reported a small increase in birth weight during the pandemic, while rates of LBW and macrosomia were unchanged [47].

Switzerland is a HIC with interesting specificities: with four national languages and the presence of alpine areas with elevated residential altitude, this country has a rich cultural, behavioural and topographical diversity. Data from the Swiss Federal Statistical Office (FSO) seem to indicate that neonatal health has not improved and even slightly worsened over the last decades, with crude LBW rates increasing from 6% to 6.5% between 2000 and 2015 [48]. Crude stillbirth rates were steadily declining until the mid-1980s, but no further improvement has been achieved since then, with a rate now oscillating between 3.5 and 4.5 per 1’000 births [49]. Birth weight and gestational age variations were recently studied at a spatial level encompassing 705 areas (called MedStat regions). Language region accounted for 23% variation of gestational age and 62% of variation in birth weight, with longer gestational duration and higher birth weight in German-speaking areas [50].

Although spatial variation in birth weight and gestational age in Switzerland has been studied, time trends and stillbirth rates have not yet been explored [50]. Furthermore, it is unclear whether recent periods of crises affected the country. The objective of our study is to explore the temporal trends in birth weight, stillbirth and PTB rates, using cross-sectional data on all births that occurred in Switzerland between 2007 and 2022. We focus on three types of crisis: an economic depression, heatwaves, and pandemics. It is important to assess whether these recent periods of crises were associated with variations in neonatal health, as adverse *in utero* environment may be associated with long-term negative health outcomes [3, 4] and even increased mortality [51]. We additionally evaluate whether crisis effect on neonatal health depends on the pregnancy trimester of exposure.

## Methods

### Data sources

This study uses routinely collected data from the FSO. The data set covers all births that occurred between 1987 and 2022 in Switzerland, at the monthly and municipality level. Due to data-anonymity rules, it was not possible to obtain data at the weekly level. We used data from 2007 because gestational age was only systematically recorded from that year onwards. The data were provided by the FSO in a fully anonymized form upon a contractual agreement. Information regarding language region, urbanity and altitude of maternal municipality, as well as the annual number of permanent Swiss residents were obtained from the FSO websites [52, 53]. According to the Human Research Act, no ethical clearance is required when working with fully anonymized governmental data [54].

### Exclusions criteria

Between 2007 and 2022, the database contained 1’517’751 births. Of these, we kept births that took place in Switzerland and for which gestational age was recorded (*n*=1’365’805). We then excluded births from mothers without a permanent resident status (*n*=43’401, comprising of short-term residents, asylum-seekers or persons working in Switzerland but living in another country), multiple pregnancies (*n*=46’666), and missing birth weight (*n*=263). Cases where either birth weight <500 grams (g) or gestational age <22 weeks were also excluded (*n*=633), since these are classified as miscarriages and not stillbirths. This implies that cases where birth weight was <500g were kept as long as gestational age was >22 weeks, because these classify as stillbirths. We also excluded missing or unlikely birth length (<20cm or ≥65cm, *n*=216) and birth weight values (<100g or ≥7 500g, *n*=11), and those with a large discrepancy between birth weight and gestational age (birth weight >2’000g and gestational age <23 weeks, or birth weight <500g and gestational age >35 weeks, *n*=21). Births from mothers aged over 50 years old were excluded as well (*n*=146), because they have much higher risks of adverse pregnancy outcomes [55]. They are also much more likely to give birth by caesarean section [56], and our dataset does not include information about delivery mode. The final dataset for the analysis of stillbirth analysis included 1’274’449 births. From this dataset, 4’863 stillbirths were excluded to explore birth weight and PTB outcomes, resulting in 1’269’586 livebirths. A flowchart of exclusion steps can be seen in Supplementary (Suppl.) Figure S1.

### Main outcome variables

We examined three outcome variables: birth weight (continuous, in grams), PTB rate (<37 completed weeks of gestation) and stillbirth rate (either gestational age ≥22 weeks or birth weight ≥500g). Birth weight is usually measured in the first hour after birth using a calibrated scale. For more than 20 years, ultrasound scan has been routinely performed to determine gestational age in almost all pregnancies in Switzerland before 12 weeks of gestation. Information on birth weight and gestational age is collected by the hospitals and midwives by filling in a form [57] which is then entered into the FSO database.

### Explanatory variables

The following covariates which were expected to be associated with the outcomes were used in the analyses. Maternal age (in years) was included as a continuous individual-level variable. In addition, we included continuous ecological variables based on the municipality in which the mother was living at the time of delivery: altitude (in meters above sea level (MASL) based on mean altitude of all residential buildings of the municipality), and mean Swiss neighbourhood index of socioeconomic position (SSEP 2.0) developed by the Institute for Social and Preventive Medicine at the University of Bern [58]. The SSEP is a high-resolution area-based index that allows studying SEP when individual-level information is missing. In our dataset, SSEP ranges from 23.6 to 86.7 index points, with higher index indicating higher SSEP. Regarding categorical variables, maternal nationality was categorized as Swiss, African, Asian, non-Swiss European, Northern American, Southern and Central American, missing and Oceanian. As the proportion of women from Oceania was very small, it was not considered a separate category and was included in the “missing” category. Civil status was categorized as single vs. married/in a registered partnership, parity as 1, 2, 3 or >3 and neonatal sex as male vs. female. At the ecological level, we included urbanity (urban vs. rural region) and language region (German/Romansh-, French- or Italian-speaking Switzerland) as categorical variables, based on maternal municipality of residence using official FSO categorisations. Language region roughly reflects cultural, behavioural, as well as dietary, smoking or alcohol consumption patterns [50].

### Exposure variables of interest

**Fig. 1:**
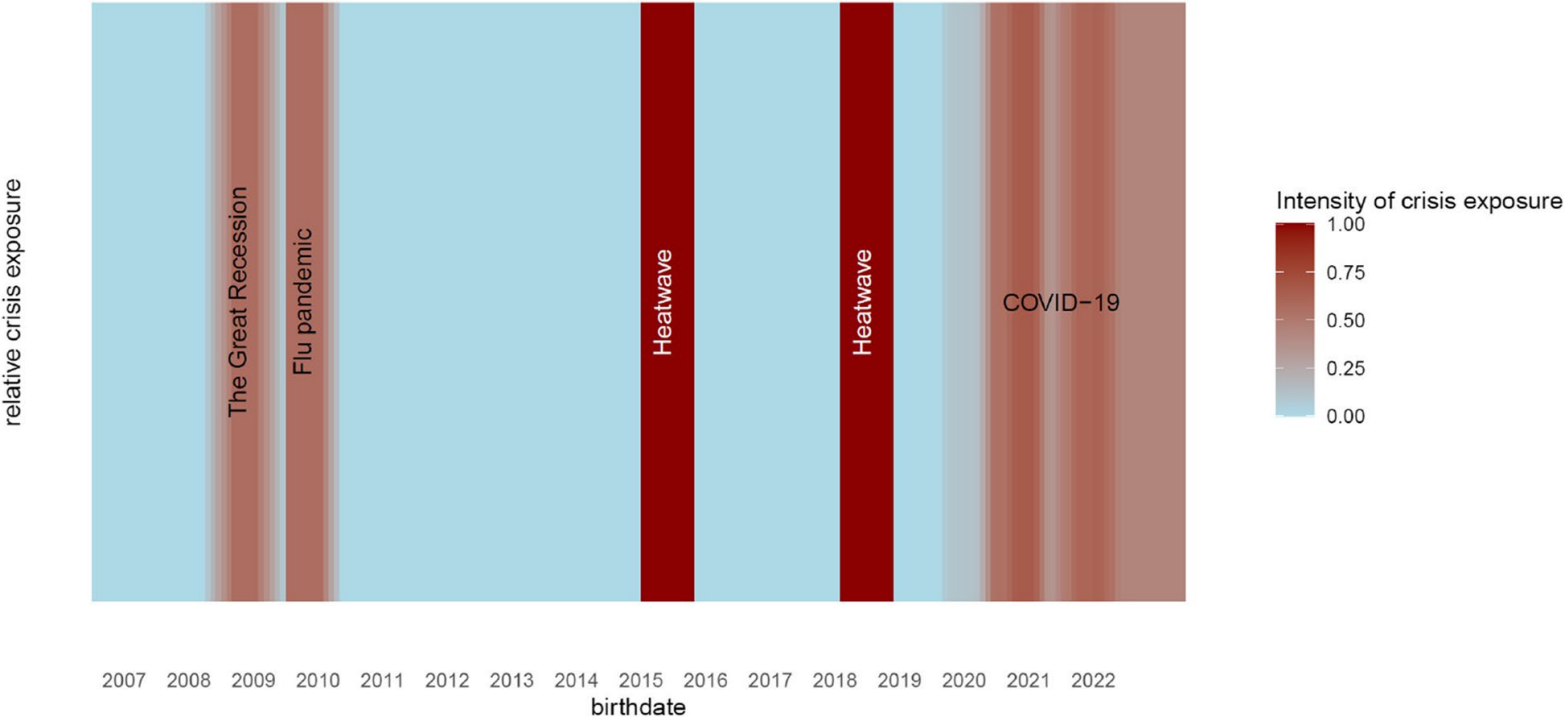
Exposure to each crisis based on birthdate. Apart from heatwave which is a dummy variable (=1 when the mother was exposed to a heatwave at any time during pregnancy), the other exposure variables represent continuous and relative exposure to the crisis per month of pregnancy

Month and year of birth were used as a continuous numerical variable from January 2007 to December 2022, adding up to a total number of 192 months. We examined four crises, namely heatwaves (2015 and 2018), the Great Recession (2008/2009), the H1N1 influenza pandemic (2009/2010) and the COVID-19 pandemic (from 2020). *Heatwaves*: the two most significant heatwaves during the studied period occurred in July 2015 and August 2018 [59]. As our temporality is only at the monthly level, we used monthly information and do not consider heatwaves’ duration nor temperatures reached. We assume that the effect of both heatwaves on neonatal health is comparable and therefore we combine the two heatwaves under the variable “heatwave exposure”. This dummy variable takes the value 1 if the mother was pregnant during a heatwave (in any month of pregnancy), and 0 otherwise. For the other three crises, which lasted longer than one month, we created relative continuous exposure variables. *Great Recession*: the economic crisis lasted six months (2008-10 to 2009-03) [60]. The number of pregnancy months overlapping with the Great Recession was summed up for each birth, then divided by gestational age at birth and normalized between 0 and 1. *Flu pandemic*: the crisis lasted 4 months (from 2009-10 until 2010-01 [61]). The flu exposure variable was constructed in the same way as for the Great Recession. The Great Recession and flu pandemic variables therefore quantify the duration of crisis exposure, relative to the total pregnancy duration. We chose exposure relative to pregnancy duration rather than absolute exposure (total number of months of pregnancy that overlapped with a crisis): infants with the shortest gestational duration were also exposed to the crisis for a smaller time period. If we do not adjust for pregnancy duration we risk seeing that the longer crisis exposure is, the less risk there is to have adverse pregnancy outcomes (stillbirth, lower birth weight, PTB). *COVID-19 pandemic*: the pandemic hit Switzerland in early 2020. As a proxy for COVID-19 exposure, we used the number of COVID-19 hospitalisations that occurred in Switzerland. Data on weekly hospitalisations from 2020-02 to 2022-12 were retrieved from the FSO [62] and aggregated at the monthly and national level. The number of hospitalisations that occurred in the country during each month of a woman’s pregnancy was summed, then divided by gestational duration and normalized between 0 and 1. The COVID-19 variable therefore quantifies the intensity of exposure per pregnancy month. The distribution of the crisis variables is displayed in Fig. 1.

### Models

#### Univariable models

We used Generalized Additive Models (GAMs) to model the variations of neonatal health over time, as this class of models allows for non-linear associations. We investigated the relationship between the exposure variable time (date of birth, modelled by a thin plate regression spline), and the outcome variables birth weight, PTB rate and stillbirth rate, separately. We used a Gaussian family for the birth weight outcome model, and a logistic family with a log link for the PTB and stillbirth outcome models. Furthermore, univariable GAMs explaining birth weight and PTB were also stratified by neonatal sex, maternal nationality, SSEP or language region of the maternal place of residence. For SSEP stratification, tertiles groups were defined: low (<54.80), medium (54.8-62.15), and high (>62.15) SSEP. The stillbirth-outcome model was not stratified, since the monthly number of stillbirth events was too low at the monthly level.

#### Multivariable models

Multivariable GAMs were used to assess the association between crisis exposure and the outcome variables while adjusting for key variables. All regression models were adjusted for the following explanatory variables: maternal age, nationality, civil status, neonatal sex, seasonality, time (date of birth), urbanity, language region, altitude and SSEP. Birth weight and PTB outcome variable models were additionally adjusted for parity (this variable was not available for stillbirth cases and not included in the stillbirth model). For the stillbirth model, maternal nationality was only categorized between Swiss or non-Swiss mothers, because of the low number of events. Variables on crisis exposure were included in all models. Since the timing of the Great Recession and the flu pandemic overlapped (see Fig. 1), we do not include both crises in the same model. The main models include the exposure variables heatwaves, Great Recession, and COVID-19 pandemic. Models with heatwaves, flu and COVID-19 pandemic are displayed in *Suppl. material*.

As the relationship between time (date of birth) and each outcome was linear in the univariable models (with a lower Bayesian information criterion -BIC- indicating a better fit), time variable was set as a linear term in the multivariable models. Maternal age and seasonality (birth month) were included as non-linear terms (implemented as thin plate regression splines and a cyclic cubic spline, respectively - see *Suppl. material* for more information on model parametrization). As gestational age is an intermediate variable on the causal path between the exposure variable (crisis) and the outcome variable (birth weight or stillbirth), adjusting or stratifying for gestational age in the birth weight or stillbirth regression models may induce bias [63–65]. Any unmeasured factor that potentially affects both gestational age and birth weight or likelihood of stillbirth (for instance, maternal comorbidities or smoking during pregnancy) may induce collider bias [64]. Therefore, we did not adjust the birth weight and the stillbirth-outcome models for gestational age, nor the PTB outcome model for birth weight. As the study sample is very large, the probability to find significant differences in our analyses is high. Reporting effect sizes allows to better assess the strength of a relationship between two variables and not only whether the association is significant or not. Thus, we report effect sizes (as Cohen’s d) along with p-values, for the linear and categorical variables. Cohen’s d values >0.1, >0.3, >0.5 are respectively considered small, moderate and large effect sizes. For the non-linear terms, we display both the graph showing their association with the outcome variable, as well as the p-value, as it is not possible to summarize the effect size with one number for these variables. As an example, the birth weight outcome model equation can be seen in the *Suppl. material (Equation E1)*.

### Trimester effect

We ran additional analyses to investigate the effects of exposure to a crisis occurring in the most vulnerable timings, i.e. first trimester (1^st^ – 3^rd^ month) or last trimester of pregnancy [66–68]. The last trimester generally corresponds to months 7-9. For the few pregnancies that lasted only two trimesters (gestational age <26 weeks, approx. 0.2% of cases), the last trimester then corresponds to pregnancy months 4 to 6. For the Great Recession and the flu, the number of months during the first trimester (0, 1, 2 or 3) which overlapped with either crisis was summed up and normalized between 0 and 1. For COVID-19, the number of hospitalisations which occurred during the first trimester was summed up, then normalized between 0 and 1. The same process was followed for last-trimester exposure. We built two models for each outcome variable: one for first-trimester exposure, and one for last-trimester exposure. Each model was adjusted for the same covariates than the main models. For an example, see *Suppl. material (Equation E2)*.

### Sensitivity analyses

Our dataset contains a non-negligible number of repeat pregnancies (*i.e.*, the same mother giving birth several times in the dataset). As we cannot adjust for this due to data confidentiality, we did a sensitivity analysis with first parities only, for birth weight and PTB outcome variables. To assess the reliability of our findings derived from models analysing birth weight as a continuous outcome, we did a sensitivity analysis using birth weight as a binary outcome: LBW (<2’500g).

All models are summarized in Table S14.

### R version and packages

R version 4.3.2 was used [69]. GAMs were built with mgcv package [70].

## Results

### Characteristics of the studied population

Between 2007 and 2022, 1’517’571 births were recorded in Switzerland. The maternal and neonatal characteristics of this population can be seen in Suppl. Table S1. After exclusion criteria (see Figure S1) we ended up with 1’274’449 singleton births in our analyses modelling stillbirth cases (whole time-period characteristics displayed in Table 1, while yearly characteristics are displayed in Tables S2-3 and Figure S2). The distribution of parity remained stable during the time period (Figure S2 A). We noticed that the share of women giving birth to singletons in German-speaking Switzerland (the country’s biggest area) decreased from 76.5% in 2007 to 71.0% in 2022, while the one from French-speaking Switzerland increased by 6.6 percentage points over the same period (Figure S2C). Most births were given by Swiss mothers, but their share declined from 65.1% to 60.7% between 2007 and 2022. Singleton neonates kept a rather constant mean birth weight, oscillating around 3 325g, with a mean gestational age of 39.3-39.4 weeks (Table S2, Figure S3D-E). Stillbirth rate slightly changed over the years without any discernible pattern (Figure S2G). The mean rate of PTB declined over the years, from 5.9% to 5.1% in 2022 (Figure S2H). LBW rate was at its lowest during the last four years under study (Figure S2I). To study birth weight and PTB outcome, we excluded stillbirth cases (*n*=4’863), resulting in the 1’269’586 livebirths (Table S3). Yearly birth rate among permanent residents was on an increasing trend between 2007 and 2016, from 9.86 to 10.50/1’000 inhabitants, but then declined until 2020. It increased back in 2021 to 10.28/1’000  inhabitants but reached its lowest rate of the whole time period in 2022 (Table S4, Figure S4).

**Table 1:** Maternal and neonatal characteristics of the analysed population (including stillbirths)

**Maternal characteristics**		**Maternal characteristics**	
**Variable**	***N*** ** = 1,274,449** ^*1*^	**Variable**	***N*** ** = 1,274,449**^1^
parity		civil status	
1	626,129 (49%)	married	982,708.0 (77.1%)
2	464,044 (36%)	single	291,741.0 (22.9%)
3	139,646 (11%)	maternal age (years)	31.4 (5.0)
>3	39,767 (3.1%)	mean SSEP	58.2 (8.6)
missing	4,863 (0.4%)	missing	205 (<0.1%)
urbanity		mean altitude	536.9 (185.5)
rural	646,138 (51%)	missing	205 (<0.1%)
urban	628,106 (49%)	**Neonatal characteristics**	
missing	205 (<0.1%)	birth weight (g)	3,325.2 (523.2)
language region		sex	
German or Romansh	911,143.0 (71.5%)	male	655,640.0 (51.4%)
French	318,553.0 (25.0%)	female	618,809.0 (48.6%)
Italian	44,548.0 (3.5%)	gestational age (weeks)	39.4 (1.8)
missing	205 (<0.1%)	gestational age category	
maternal nationality		term	1,204,114.0 (94.5%)
Switzerland	782,480.0 (61.4%)	preterm (<37 weeks)	70,335.0 (5.5%)
Europe	373,904.0 (29.3%)	living status	
Asia	44,915.0 (3.5%)	livebirth	1,269,586.0 (99.6%)
Africa	37,187.0 (2.9%)	stillbirth	4,863.0 (0.4%)
Southern and Central America	22,910.0 (1.8%)	birth weight category	
Northern America	7,474.0 (0.6%)	normal BW	1,214,013.0 (95.3%)
missing or Oceanian	5,579.0 (0.4%)	low birth weight (<2'500g)	60,436.0 (4.7%)

^1^n (%); Mean (SD)

### Neonatal health trend over the years and under crises exposure

#### Birth weight

**Fig. 2:**
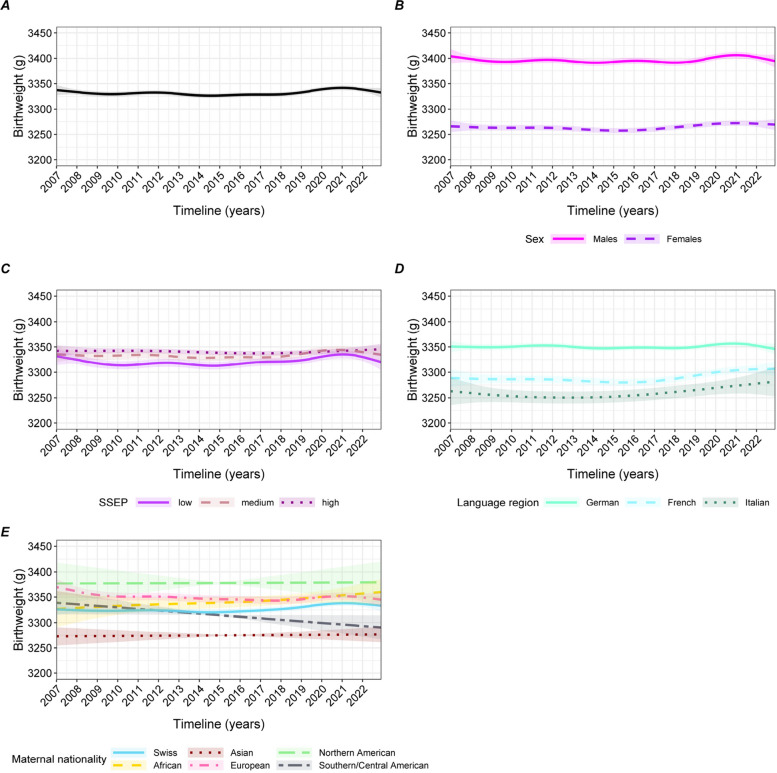
Association between birth weight and birth date from a GAM. All models are univariable. **A** unstratified. The other graphs are stratified by: sex (**B**), SSEP (**C**), language-region (**D**), and maternal nationality (**E**)

The univariable GAM shows that birth weight varied very little between 2007 and 2022 (Fig. 2A). Stratifications by sex, SSEP, language region and maternal nationality (Fig. 2A-E) display similar trends: birth weight was virtually constant over the time period. During periods of crises, we do not see any shift in birth weight, except for a minor upward trend towards the end of the time trend, which partly overlaps with the COVID-19 pandemic. However, we see systematic subgroup differences. For instance, stratification by SSEP reveals that birth weight oscillated around slightly different values depending on SSEP tertile: 3’320, 3’340 and 3’350g for the lowest, medium and highest tertiles, respectively (Fig. 2C). Babies in German-speaking Switzerland were heavier than their counterparts in French- and Italian-speaking regions (Fig. 2D). Table 2 shows the adjusted results of the birth weight GAM. When mothers were exposed to a heatwave or to COVID-19 during pregnancy, birth weight was slightly higher (9.0g [95% confidence interval (CI) 6.0 to 12.0]) and 11.7g [95%CI 5.5 to 17.9], respectively), whereas it was lower when the mother was exposed to the Great Recession (-9.6g [95%CI -18.3 to -1.0]). However, Cohen’s d for all crises variables is virtually 0, indicating that the effect sizes were very small. Male neonatal sex and maternal increasing parity were associated with higher birth weight, while maternal nationality also has an influence. Each 100 MASL increase in altitude was associated with lower birth weight. The association between birth weight and the smoothed variables can be seen in Figure S5. Youngest and oldest (<20 or >40 years old) maternal age was associated with a lower birth weight compared to middle-aged mothers (Figure S5A). The LBW outcome models are mostly consistent with these findings (Figures S11, 12, Table S9), depicting only a very small decline in the rate of LBW from 2015 (Figure S11A), while infants born in municipalities belonging to the lowest SSEP tertile also had the highest LBW rate (Figure S11C). LBW rate was increasing through time among women from Southern and Central America (Figure S11E); this is also reflected by the declining birth weight trend in Fig. 2E. However, very few women were from these regions (<2%, see Table 1). Interestingly, we also find that there was a 0.5% increase and subsequent decrease of LBW rate in French-speaking Switzerland, between 2007 and 2022 (Figure S11D). Regarding the crises, only heatwave exposure was significantly associated with a lower LBW risk (Table S9).

**Table 2:** Birth weight linear regression GAM (model 1.1)

			**95% CI (g)**			
**Variable**	**Category**	**beta**	**lci**	**uci**	**d**	***p*value**
Heatwave (ref: 0)	1	9.02	6.02	12.02	0.01	<0.0001
Great Recession (continuous)		-9.63	-18.25	-1.01	0.00	0.03
COVID (continuous)		11.67	5.47	17.88	0.00	<0.001
Time (by month)		0.04	0.01	0.06	0.00	<0.01
SSEP (/10 points)		4.55	3.43	5.68	0.01	<0.0001
Altitude (/100m)		-6.99	-7.50	-6.48	0.02	<0.0001
Parity (ref: 1)	2	123.41	121.46	125.36	0.11	<0.0001
	3	163.35	160.37	166.33	0.10	<0.0001
	>3	188.44	183.28	193.60	0.06	<0.0001
Sex (ref: male)	Female	-132.26	-133.99	-130.52	0.13	<0.0001
Urban (ref: rural)	Urban	2.07	0.25	3.89	0.00	0.03
Language region (ref: German)	French	-52.98	-55.09	-50.87	0.04	<0.0001
	Italian	-89.20	-94.06	-84.33	0.03	<0.0001
Maternal nationality (ref: Swiss)	Africa	11.90	6.65	17.15	0.00	<0.0001
	Asia	-58.83	-63.60	-54.06	0.02	<0.0001
	Europe	27.09	25.11	29.07	0.02	<0.0001
	Northern America	71.72	60.35	83.09	0.01	<0.0001
	Southern/Central America	5.58	-0.99	12.16	0.00	0.10
Civil status (ref: married)	Single	-37.88	-40.03	-35.73	0.03	<0.0001
**Smooth variables**						
Maternal age (years)						<0.0001
Seasonality (month)						<0.01
*n*=1’263’853						

95%CI: 95% Confidence interval, lci: lower confidence interval, uci: upper confidence interval, d: Cohen’s d. Cohen’s d >0.1, >0.3, >0.5 are respectively considered small, moderate and large effect sizes. Great Recession and COVID-19 exposure variables are relative to pregnancy duration (values between 0 and 1). SSEP scale goes from 23.6 to 86.7, by 10 points increase

#### Preterm birth

**Fig. 3:**
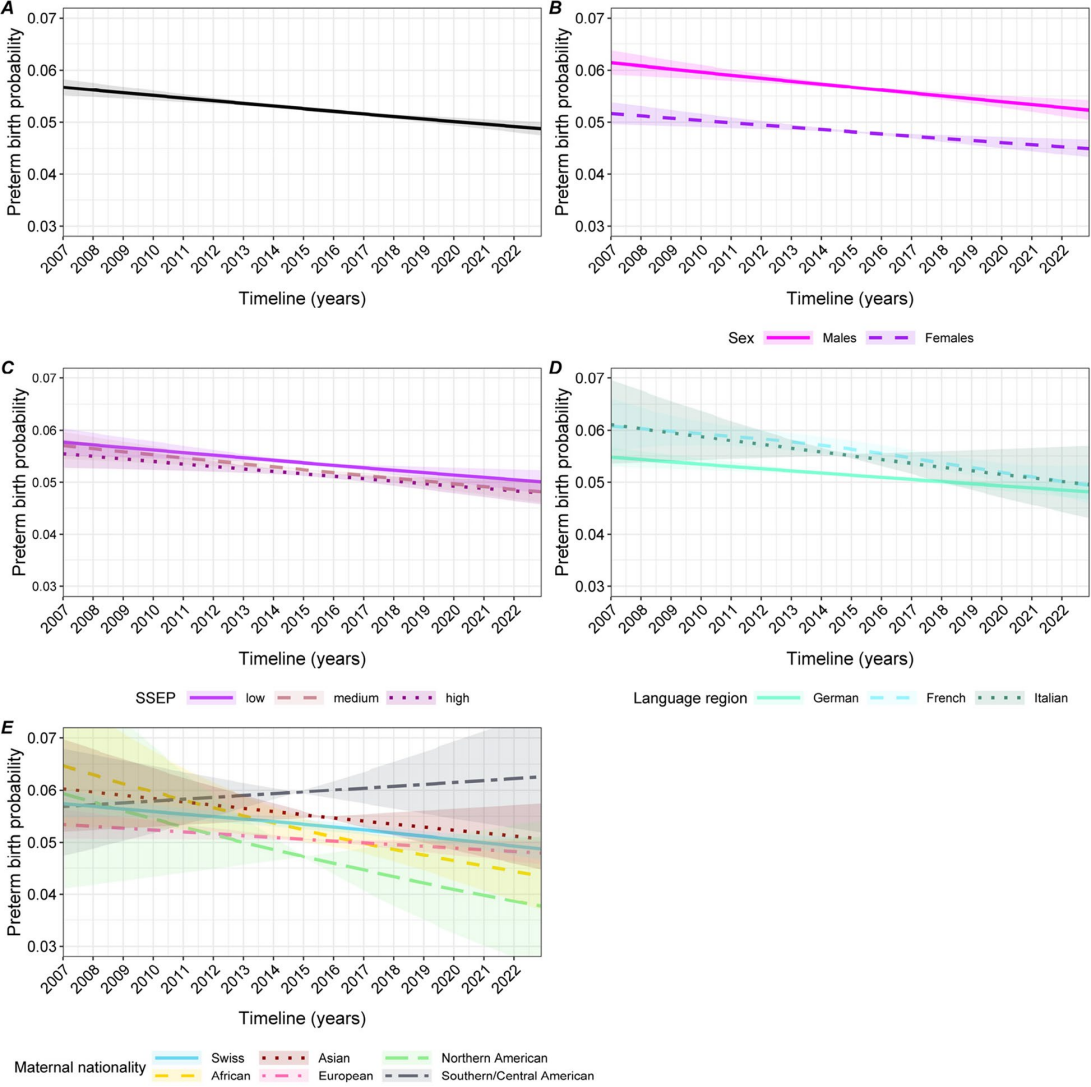
Association between preterm birth rate and birthdate from a GAM. All models are univariable.** A**: unstratified. The other graphs are stratified by: sex (**B**), SSEP (**C**), language-region (**D**), and maternal nationality (**E**)

Preterm birth univariable GAM displays a small and continuous downward trend through time, from 5.7% in 2007 to 4.9% in 2022 (Fig. 3A). This trend is consistent in all stratified GAMs (Fig. 3B-E), except for an increasing PTB risk among babies from Southern and Central American mothers (Fig. 3E). As for birth weight, systematic differences remain over time, with for instance females being less likely to be born before term than males (Fig. 3B).

In the multivariable GAM, exposure to a heatwave is associated with a lower probability of PTB, with an odds ratio (OR) of 0.85 [95%CI 0.82 to 0.87] and a Cohen’s d of 0.09, depicting a small effect size (Table 3). Exposure to the Great Recession or to COVID-19 are not associated with a change in PTB risk. Concerning the other covariates that we adjusted on, male neonatal sex, first parities and single mothers have higher PTB risk. Youngest and oldest maternal age (<20 or >40 years old) (Figure S6 A) are associated with higher risks of PTB.

**Table 3:** Preterm birth logistic regression GAM (model 2.1)

**Variable**	**Category**	**OR**	**95% CI**		**d**	***p*value**
			**lci**	**uci**		
Heatwave (ref: 0)	1	0.85	0.82	0.87	0.09	<0.0001
Great Recession (continuous)		1.04	0.97	1.13	0.02	0.27
COVID (continuous)		0.98	0.92	1.03	0.01	0.40
Time (by month)		1.00	1.00	1.00	0.00	<0.0001
SSEP (/10 points)		0.96	0.95	0.97	0.02	<0.0001
Altitude (/100m)		1.00	1.00	1.00	0.00	1.00
Parity (ref: 1)	2	0.67	0.66	0.69	0.22	<0.0001
	3	0.68	0.67	0.70	0.21	<0.0001
	>3	0.78	0.74	0.81	0.14	<0.0001
Sex (ref: male)	Female	0.84	0.83	0.85	0.10	<0.0001
Urban (ref: rural)	Urban	0.95	0.94	0.97	0.03	<0.0001
Language region (ref: German)	French	1.05	1.03	1.07	0.03	<0.0001
	Italian	1.00	0.96	1.05	0.00	0.87
Maternal nationality (ref: Swiss)	Africa	1.01	0.96	1.05	0.00	0.83
	Asia	1.07	1.03	1.12	0.04	<0.01
	Europe	0.95	0.93	0.97	0.03	<0.0001
	Northern America	0.85	0.76	0.94	0.09	<0.01
	Southern/Central America	1.09	1.03	1.15	0.05	<0.01
Civil status (ref: married)	Single	1.12	1.10	1.14	0.06	<0.0001
**Smooth variables**						
Maternal age (years)						<0.0001
Seasonality (month)						<0.0001
*n*=1’263’853						

*95%CI* 95% Confidence interval, *OR* Odds-ratio, *lci* lower confidence interval, *uci* Upper confidence interval, d: Cohen’s d. Cohen’s d >0.1, >0.3, >0.5 are respectively considered small, moderate and large effect sizes. Great Recession and COVID-19 exposure variables are relative to pregnancy duration (values between 0 and 1). SSEP scale goes from 23.6 to 86.7, by 10 points increase

#### Stillbirth

**Fig. 4:**
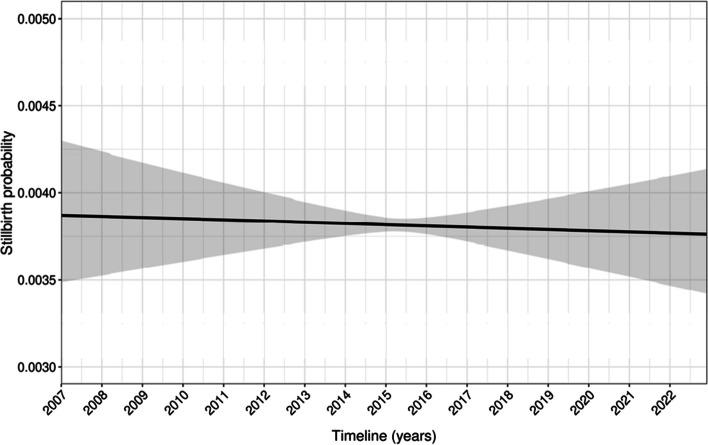
Association between stillbirth rate and birthdate from a GAM. This model is unstratified and unadjusted

There is no evidence that stillbirth rate significantly varied through time (3.9‰ to 3.8‰) (Fig. 4).

Exposure to a heatwave is associated with a lower risk of stillbirth: OR 0.77 [95%CI 0.69 to 0.86], but we do not find evidence that exposure to the Great Recession or to the COVID-19 pandemic had a significant impact on stillbirth (Cohen’s d are very close to 0, Table 4). Higher SSEP was associated with a lower stillbirth probability, while infants from mothers who were single, living in French-speaking Switzerland or who did not have the Swiss nationality had a higher risk of being stillborn (Table 4).

**Table 4:** Stillbirth logistic regression GAM (model 3.1)

**Variable**	**Category**	**OR**	**95% CI**		**d**	***p*value**
			**lci**	**uci**		
Heatwave (ref: 0)	1	0.77	0.69	0.86	0.14	<0.0001
Great Recession (continuous)		1.16	0.88	1.52	0.08	0.28
COVID (continuous)		1.14	0.93	1.39	0.07	0.21
Time (by month)		1.00	1.00	1.00	0.00	0.18
SSEP (/10 points)		0.90	0.87	0.93	0.06	<0.0001
Altitude (/100m)		0.99	0.97	1.00	0.01	0.13
Sex (ref: male)	Female	1.01	0.95	1.07	0.01	0.72
Urban (ref: rural)	Urban	1.06	0.99	1.12	0.03	0.08
Language region (ref: German)	French	1.14	1.06	1.22	0.07	<0.001
	Italian	0.94	0.80	1.10	0.03	0.44
Maternal nationality (ref: Swiss)	Not Swiss	1.13	1.06	1.19	0.07	<0.001
Civil status (ref: married)	Single	1.38	1.29	1.47	0.18	<0.0001
**Smooth variables**						
Maternal age (years)						<0.0001
Seasonality (month)						0.70
*n*=1,270,214						

*95%CI* 95% Confidence interval, *OR* Odds-ratio, *lci* Lower confidence interval, *uci* Upper confidence interval, d: Cohen’s d. Cohen’s d >0.1, >0.3, >0.5 are respectively considered small, moderate and large effect sizes. Great Recession and COVID-19 exposure variables are relative to pregnancy duration (values between 0 and 1). SSEP scale goes from 23.6 to 86.7, by 10 points increase

### Neonatal health during crises: trimester effect

Higher exposure to the COVID-19 pandemic during the last trimester was associated with an increase in birth weight (16.8 g, [95%CI 10.7 to 23.0], Table 5). However, heatwave exposure during the last trimester was associated with a lower birth weight (-10.8 g, [95%CI -15.8 to -5.9], Table 5): this is inconsistent with the main birth weight model (Table 2). Still, Cohen’s d effect sizes are rounded to 0, showing that although some associations are significant, their effect sizes are very small. Using LBW as an outcome, we find that only last-trimester exposure to COVID-19 was significantly associated with a slightly lower risk of LBW (Table S10), thus matching with the higher mean birth weight reported in Table 5. The aforementioned finding of a lower PTB risk when the mother was exposed to a heatwave during pregnancy (Table 3) may be mediated by first-trimester exposure (OR 0.95 [95%CI 0.91 to 1.00]), while last-trimester exposure to a heatwave did not appear to affect the risk of PTB (Table 5). On the contrary, intensified exposure to the COVID-19 pandemic during the first three months of pregnancy was associated with a slightly higher PTB risk (OR 1.06 [95%CI 1.00 to 1.12]). Last-trimester exposure to the COVID-19 pandemic was associated with a higher stillbirth risk, with an OR of 1.24 [95%CI 1.02 to 1.50] and a Cohen’s d of 0.12, denoting a small effect.

**Table 5:** Trimester effect of crises on birth weight, preterm birth and stillbirth

		**Birthweight**					**Preterm birth**					**Stillbirth**				
**Trimester**	**Crisis**	**beta (g)**	**lci**	**uci**	**d**	***p*value**	**OR**	**lci**	**uci**	**d**	***p*value**	**OR**	**lci**	**uci**	**d**	***p*value**
**First**	**Heatwave**	0.20	-4.91	5.32	0.00	0.94	0.95	0.91	1.00	0.03	0.04	0.97	0.82	1.15	0.02	0.74
	**Great Recession**	0.15	-5.59	5.89	0.00	0.96	0.98	0.93	1.03	0.01	0.42	0.98	0.81	1.18	0.01	0.80
	**COVID-19**	-6.27	-12.17	-0.36	0.00	0.04	1.06	1.00	1.12	0.03	0.03	1.04	0.86	1.26	0.02	0.68
**Last**	**Heatwave**	-10.84	-15.77	-5.92	0.00	<0.001	1.02	0.98	1.07	0.01	0.31	0.99	0.85	1.17	0.00	0.94
	**Great Recession**	-3.17	-9.13	2.79	0.00	0.30	1.02	0.96	1.07	0.01	0.55	1.10	0.91	1.32	0.05	0.32
	**COVID-19**	16.82	10.66	22.97	0.00	<0.0001	0.98	0.93	1.04	0.01	0.54	1.24	1.02	1.50	0.12	0.03

*OR* Odds-ratio, *lci* Lower confidence interval, *uci* Upper confidence interval, d: Cohen’s d. Corresponding models are model 1.1.A and 1.1.B (birth weight), model 2.1.A and 2.1.B (preterm birth), and model 3.1.A and 3.1.B (stillbirth). Cohen’s d >0.1, >0.3, >0.5 are respectively considered small, moderate and large effect sizes

Results of the models using the 2009 flu pandemic instead of the Great Recession can be seen in Tables S5-8 and Figures S8-10 and indicate that being exposed to the flu slightly reduced the risk of PTB in general (OR 0.89 [95%CI 0.83 to 0.95], Table S6), and in the first as well as last trimesters (Table S8).

### Sensitivity analysis: first parities

Using only first parities in the models (Tables S11-13, Figures S13-14), the results were consistant with the main models including all parities.

## Discussion

We used nationwide cross-sectional data to assess changes in neonatal health outcomes over the years 2007-2022 and during periods of crises in Switzerland. Since 2007, birth weight and the rate of stillbirth were only subject to minor changes, while the rate of preterm birth constantly declined. Our stratified models by neonatal sex, maternal nationality, SSEP and language region show systematic differences between categories but display similar trends. Exposure to crises was associated with different effects on neonatal health, depending on the type of crisis.

### Neonatal health trends

The World Health Organization (WHO) reported that global PTB rates were stable from 2010 to 2020, with values around 10% even among HICs [16, 25]. However, PTB rates were decreasing between 2006 and 2014 in the US but also in Norway, as we report here for Switzerland [24]. While changes in the way gestational age is reported can contribute to PTB rate variation [23, 71, 72], obstetric interventions can also influence it. In Switzerland, caesarean-section rate remained consistently higher than 32% between 2007 and 2022 [73], well above the 10% rate recommended by the WHO [74]. However, the rate varied too little to fully account for the declining PTB trend. In 2017, assisted reproductive technology (ART) changed with the introduction of a single embryo transfer [75]. This reduced the occurrence of multiple births, thus decreasing the number of PTB associated with multiple pregnancies [75]. Although we focus on singletons, these changes might have affected their outcomes as well, increasing the number of singleton births born through ART.

Our result on stillbirth rate mirrors data already-published from the FSO: only minor variations of unadjusted rates are reported between 2007 and 2022 [49]. While some reports point towards decreasing trends between 2000 and 2019 in Western Europe and other HICs [27, 28], this decline has recently slowed down [29]; stillbirth rate was even stable in Germany between 2009 and 2012 [76]. Stillbirth rate might be stagnating in Switzerland because of increasing maternal age [29] or prevalence of maternal comorbidities (such as obesity and diabetes), impeding stillbirth rate of declining further.

Birth weight evolution varied a lot across countries around the turn of the 21^st^ century. Some HICs experienced declining birth weight [17–20], but it increased in England [21], while Norway and Sweden exhibited an unexplained increase and then decrease of birth weight [19]. We see that birth weight was mostly constant in Switzerland during the studied period.

### Crises

Crises such as economic depressions and pandemics have a direct burden on the population’s health in terms of mortality and morbidity, but also create social and economic disruptions and traumatic experiences. The highest-risk crises are without doubt armed conflicts and natural disasters, increasing stress among pregnant individuals through life-threatening events and having an uncertain future. These types of crises have also been associated with higher rates of LBW [30]. This paper focuses on population-level crises, and especially on their potential indirect impact on pregnancies, through stress exposure. In our univariable models, we do not see shifts in birth weight, nor in the rates of PTB or stillbirth during any of the crises investigated. When we consider crisis-exposure in more details using multivariable models, exposure to the Great Recession or to COVID-19 were not associated with variations in PTB nor with stillbirth probabilities. However, when we separate the exposure per pregnancy trimester, COVID-19 slightly increased PTB risk for first trimester exposure, and stillbirth risk for last-trimester exposure.

The impact of an economic crisis on neonatal health depends on the initial family financial situation. For instance, an individual-level study found an important association between inadequate employment and birth weight decrease [37]. In Switzerland, the unemployment rate almost doubled between the beginning of the Great Recession and the end of 2009, from 2.4% to 4.4% [77]. If the crisis only impacted already socioeconomically disadvantaged families, we might not be able to see an important effect overall. We control for SSEP, an area-based information, but lack individual information on employment status. The Great Recession was associated with more important effects in countries already socioeconomically disadvantaged. In Portugal, a higher LBW prevalence was noted [35], while in Greece, crude stillbirth, infant and child mortality rates gradually increased during the Great Recession [36]. Given that Switzerland ranks among the highest income countries, it was likely less affected by the economic crisis.

Multiple studies reported decreased PTB rates during the COVID-19 pandemic [78], including a meta-analysis on 52 million births from 26 countries [79]. However, some suggest that this may be due to reporting biases, because the association was no longer significant for adjusted rates [78]. In Switzerland, PTB rate slightly declined in 2020 [80], but the year 2019 was already displaying lower rates, indicating that the reduced PTB odds in 2020 is unlikely to be solely attributable to the pandemic. In addition, the previously-mentioned meta-analysis did not identify any change in PTB rate during the COVID-19 lockdown in Switzerland [79]. Our results are thus consistent with the literature and, with the use of individual-level information, add further evidence suggesting that the COVID-19 pandemic only had, if any, a small effect on PTB rates.

Separating the exposure by pregnancy trimesters, we find a higher risk of stillbirth for exposure to COVID-19 during the last trimester, with an OR of 1.24 [95%CI 1.02 to 1.50]. The associated Cohen’s d is 0.12, which is higher than any d’s of the other crises among the trimesters-effect models, but still denotes a very small effect size. A large meta-analysis mentions no change in stillbirth rate during the pandemic (OR 1.08, [95%CI 0.94 to 1.23]), *n*= 21 studies) [78]. Few studies separated analyses by pregnancy trimester. In the Indian state of Bihar, stillbirth rate increased in a dose-relationship manner with the number of last trimester months that occurred during the pandemic peak [81]. Whether our finding of a slight increase in stillbirth rate results from maternal severe acute respiratory syndrome coronavirus 2 (SARS-CoV-2) infection or increased stress exposure is uncertain. Although the literature on SARS-CoV-2 vertical transmission is conflicting, maternal infection has been reported to cause placental inflammation. A multi-national study linked 68 stillbirths with maternal SARS-CoV-2 infection, and identified placental abnormalities that could have caused placental insufficiency and foetal death [82]. Maternal stress might be another explanation: shortage of obstetric staff and reduced prenatal care, including mothers postponing visits due to fear of getting infected, might have played a role in stillbirth rate, as Khalil et al. suggest [83].

When women were exposed to COVID-19 during pregnancy, birth weight was higher by 11.7g [95%CI 5.5 to 17.9]). Birth weight was higher for last trimester (16.8g [95%CI 10.7 to 23.0]) but not first trimester exposure (-6.3g [95%CI -12.2 to -0.4]). Two meta-analyses reported small birth weight increases during the pandemic (17g [95%CI 7 to 28] [78] and 15g [95%CI 10 to 20] [47]). In Denmark, a similar increase during the first lockdown was noted among singleton term births [84]. The sex- and gestational age- adjusted increase was of 17g [95%CI 3 to 31]. Increasing birth weight can indeed be due to longer pregnancy duration, but we did not adjust the birth weight-outcome model for gestational age due to potential collider bias [63–65]. To assess whether our result of a higher birth weight could be linked to longer gestational duration, we display yearly rates of term births for each gestational week: babies did not have longer gestation during the COVID-19 years (Table S15). This supports the idea that higher birth weight among mothers exposed to COVID-19 is not due to longer gestation. Our results align closely with existing literature, showing a similar birth weight increase. The implications of such a small increase are uncertain and do not always imply changes in LBW odds [78]. However, we here found that COVID-19 exposure during the last trimester was also associated with a slight reduction of LBW risk (OR 0.94 [95%CI 0.88-1.00].

Indirect effects of the pandemic may be negative, *i.e.* through stress exposure or dietary changes, associated with a more sedentary life during lockdowns. Home office was frequent during the pandemic, and might have induced changes in nutrition and a lack of exercise, which could explain the slight increase in birth weight. Furthermore, pregnant women’s mental health was reported to have worsened during the pandemic [45, 85], including in Switzerland [46]. On the contrary, pandemic exposure could also have had positive effects: Switzerland had a light lockdown and was less economically affected compared to surrounding countries: there might even have been an improved work-life balance and hygiene, and a limited exposure to pollution. Still, some women of our dataset were directly affected through SARS-CoV-2 infection, with well-documented increased risks of maternal mortality, PTB and LBW [44, 86, 87]. Thus, the effects of the pandemic on maternal and neonatal health may be opposite [84]: if there were both negative and positive effects of the mitigation measures put into place, as well as negative effects of maternal infection, they might have compensated each other. This could explain the overall absence of association between the COVID-19 pandemic and the neonatal health outcomes we investigated, except for a small stillbirth risk increase. COVID-19 vaccination was extended to all pregnant women in September 2021 in Switzerland [88] and might have prevented severe complications and influenced PTB and stillbirth rates. Unfortunately, there exist no vaccination coverage data among pregnant women. Those who gave birth in 2022 might have been vaccinated during pregnancy, which limits our interpretation. We also observe a birth rate decline in the beginning of 2022. This downturn in births was reported in multiple countries, starting in January 2022 [89]. The effect of crises on fertility rates requires further study and was not the objective of the current study.

Exposure to heatwaves during pregnancy was associated with higher birth weight and lower probabilities of PTB and stillbirth. However, exposure during the first trimester was not associated with birth weight, while last-trimester exposure was linked to lower birth weight. The discrepancy between the main model and the trimester-effect models might be explained by the structure of our data: using monthly-level data may not allow investigating heatwave exposure in detail, since a heatwave usually lasts only a few days. Moreover, some pregnancies which started or ended during a heatwave month might have actually not overlapped with the heatwave event. These pregnancies would have thus been miscategorized as “exposed to a heatwave”; this should however concern very few pregnancies. Most studies describe positive associations between heat exposure and PTB [31], but a meta-analysis concluded that the evidence of reduced birth weight is limited [34]. Few studies have focused on stillbirth, but their majority report an increased risk [31, 32]. In low- and middle-income countries, already experiencing the highest PTB and stillbirth rates, higher temperatures were associated with increased risks of both outcomes [33]. As the frequency and severity of heatwaves is likely to increase in the near future, it is crucial to continue to assess their effect on foetal health. The structure of our data does not allow us to conclude on the effect of heatwave exposure on neonatal health.

The effects of the covariates on which we adjusted the analyses are consistent with the literature, with higher risks of PTB and stillbirth when maternal age increases [13, 90], and primiparous mothers having lighter babies [91]. Birth weight was reduced in French- and Italian-speaking regions of Switzerland, and also at elevated altitudes, as shown previously [50]. Babies born from non-Swiss mothers had a higher risk of stillbirth: Turkish mothers living in Switzerland were previously reported to have a 30% higher stillbirth risk [92]. We also show that mothers from Asian and Southern/Central American regions have higher risks of PTB, while those from Europe and Northern America have a lower risk: maternal ethnicity is known to affect neonatal health, probably more through an interplay of environmental and sociodemographic parameters than through genetic determinants [93–95]. However, we use nationality as a crude proxy for ethnicity, which is limited. Interestingly, we highlight an effect of civil status on neonatal health, increasing stillbirth risk by 38%. Similarly, neonatal and infant mortality were recently reported to be much higher among unmarried mothers in Switzerland [65]. Important covariates that we could not adjust for are maternal comorbidities and smoking, known to be associated with lower birth weight [96] and stillbirth risk [90]. Our data does not include information on maternal overweight (body mass index (BMI) ≥25) and obesity (BMI ≥30) which are major risk factors for stillbirth in HICs [90].

Our study is thus limited by the structure of our dataset and available covariates. Regarding COVID-19, we cannot identify mothers who were infected by the virus: it is thus not possible to disentangle the direct effect of maternal infection from the indirect effect of stress and mitigation measures. We used hospitalisation cases as a proxy of COVID-19 exposure, regardless of waves and circulating viral strains. Regarding spatial differences, it was mostly during the first COVID-19 wave that the Cantons were differently affected [97]. However, few people were actually infected during this wave [98], and the subsequent waves had similar magnitudes geographically [97]. Furthermore, at the population level, we expect the waves to have the same effect on pregnant mothers in terms of stress. The choice of outcomes could also be discussed: the PTB variable does not differentiate between spontaneous and iatrogenic PTB, and these have different determinants [15].

The robustness of our results relies on the very large sample size, covering nation-wide singleton births at the individual level. Our birth weight- , LBW- and PTB-stratified analyses display consistent results. We can thus be confident that birth weight was stable through time in almost every subgroup assessed, and that PTB declined during the same period. Our sensitivity analyses focusing on first parities, and our analyses with the flu pandemic exposure instead of the Great Recession, enhance the reliability of our findings.

## Conclusion

We have shown that birth weight and the rate of stillbirth varied very little in Switzerland between 2007 and 2022. However, we see a clear PTB rate decline over that period of about 1%. Similar trends are found in all investigated subgroups. Overall, effect sizes of all investigated associations between crises and neonatal outcomes are small. With regards to our results, Switzerland, being among the highest income countries globally, appears resilient to economic crises and pandemics at the national level, compared to other European countries. However, the COVID-19 pandemic impact on maternal and neonatal health might have been double-edged. Although the negative impact of maternal infection on pregnancy outcomes is supported by the literature, we cannot rule out that stress and economic constraint also affected neonatal health among the most vulnerable subgroups of Switzerland.

### Supplementary Information

**Supplementary Material 1.** 

## Data Availability

The dataset that support the findings of this study are available from FSO but restrictions apply to the availability of these data, which were used under license for the current study, and so are not publicly available. The codes used in this study are publicly available in the following Github online repository: https://github.com/MathildeLV/BevNat.

## Abbreviations

ART: Assisted reproductive technology
BMI: Body mass index
CI: Confidence interval
COVID-19: Coronavirus disease 2019
FSO: Swiss Federal Statistical Office
g: Grams
GAM: Generalized Additive Model
HIC: High-income country
LBW: Low birth weight
MASL: Meters above sea level
OR: Odds ratio
PTB: Preterm birth
SARS-CoV-2: Severe acute respiratory syndrome coronavirus 2
sd: Standard deviation
SEP: Socio economic position
SSEP: Swiss neighbourhood index of socioeconomic position
Suppl.: Supplementary
WHO: World Health Organization

## References

[1] WHO. Goals. 3. Ensure healthy lives and promote well-being for all at all ages. 2015. https://sdgs.un.org/goals/goal3.

[2] WHO. Global Nutrition Monitoring Framework: Operational for Tracking Progress in Meeting Targets for 2025. 2017.

[3] Barker D (1990). The fetal and infant origins of adult disease. BMJ.

[4] Barker DJP (1997). Fetal nutrition and cardiovascular disease in later life. Br Med Bull.

[5] Ward, W. P. Birth Weight as an Indicator of Human Welfare. in The Oxford Handbook of Economics and Human Biology 621–632 (Oxford University Press). 2016. 10.1093/oxfordhb/9780199389292.013.33.

[6] Wilcox AJ, Skcerven R (1992). Birth Weight and Perinatal Mortality: The Effect of Gestational Age. Am J Public Health.

[7] Mc Intire D, Bloom S, Casey B, Leveno K (1999). Birth weight in relation to morbidity and mortality among newborn infants. N Engl J Med.

[8] Richardson AN, Pollak EA, Williams D, Smith MA (2010). Intrauterine Infection. Comprehensive Toxicol Sec Edit.

[9] Goldenberg RL, Culhane JF, Johnson DC (2005). Maternal infection and adverse fetal and neonatal outcomes. Clin Perinatol.

[10] McClure EM, Dudley DJ, Reddy UM, Goldenberg RL (2010). Infectious causes of stillbirth: a clinical perspective. Clin Obstet Gynecol.

[11] World Health Organization. Preterm birth. https://www.who.int/news-room/fact-sheets/detail/preterm-birth (2023).

[12] Goldenberg RL, Culhane JF, Iams JD, Romero R (2008). Epidemiology and causes of preterm birth. Lancet.

[13] Fuchs F, Monet B, Ducruet T, Chaillet N, Audibert F. Effect of maternal age on the risk of preterm birth: a large cohort study. PLoS One. 13(1):e0191002. 10.1371/journal.pone.0191002 PMC5791955 29385154

[14] van Winden T (2023). Impact of ethnicity and neighborhood deprivation on preterm birth: How does urban living play a role?. Eur J Obstet Gynecol Reprod Biol.

[15] Mensah NA (2023). Examining recent trends in spontaneous and iatrogenic preterm birth across race and ethnicity in a large managed care population. Am J Obstet Gynecol.

[16] World Health Organization. Born Too Soon: Decade of Action on Preterm Birth. 2023.

[17] Oken, E. Secular trends in birthweight. in Nestle Nutrition Institute Workshop Series. 2013; 71: 103–114 (S. Karger AG,). 10.1159/000342576 23502144

[18] Kato N, Sauvaget C, Yoshida H, Yokoyama T, Yoshiike N (2021). Factors associated with birthweight decline in Japan (1980–2004). BMC Pregnancy Childbirth.

[19] Carlsen EØ (2020). Stumped by the Hump: The Curious Rise and Fall of Norwegian Birthweights, 1991–2007. Epidemiology.

[20] Fuster, V. & Santos, C. Determinants of Birth Weight in Portugal: 1988 to 2011. J Biol Clin Anthropol. 2016; 73. 10.1127/anthranz/2015/0541 26754741

[21] Ghosh RE, Berild JD, Sterrantino AF, Toledano MB, Hansell AL (2018). Birth weight trends in England and Wales (1986–2012): babies are getting heavier. Arch Dis Child Fetal Neonatal Ed.

[22] Zeitlin J (2013). Preterm birth time trends in Europe: A study of 19 countries. BJOG.

[23] Morken NH, Källen K, Hagberg H, Jacobsson B (2005). Preterm birth in Sweden 1973–2001: Rate, subgroups, and effect of changing patterns in multiple births, maternal age, and smoking. Acta Obstet Gynecol Scand.

[24] Richards JL (2016). Temporal trends in late preterm and early term birthrates in 6 high-income countries in North America and Europe and association with clinician-initiated obstetric interventions. JAMA.

[25] Blencowe H (2012). National, regional, and worldwide estimates of preterm birth rates in the year 2010 with time trends since 1990 for selected countries: a systematic analysis and implications. Lancet.

[26] Schneider EB (2017). Fetal health stagnation: Have health conditions in utero improved in the United States and Western and Northern Europe over the past 150 years?. Soc Sci Med.

[27] Hug L. et al. Articles Global, Regional, and National Estimates and Trends in Stillbirths from 2000 to 2019: A Systematic Assessment. 2021; 398. www.thelancet.com. 10.1016/S0140-6736(21)01112-0 PMC8417352 34454675

[28] Norris T, Manktelow BN, Smith LK, Draper ES (2017). Causes and temporal changes in nationally collected stillbirth audit data in high-resource settings. Semin Fetal Neonatal Med.

[29] Kayode GA, et al. Temporal trends in stillbirth over eight decades in England and Wales: a longitudinal analysis of over 56 million births and lives saved by improvements in maternity care. J Glob Health. 2022;12:04072. 10.7189/jogh.12.04072 PMC9480862 36112509

[30] Keasley J, Blickwedel J & Quenby S. Adverse effects of exposure to armed conflict on pregnancy: a systematic review. BMJ Global Health. 2017; 2. 10.1136/bmjgh-2017-000377. 10.1136/bmjgh-2017-000377 PMC5706483 29333283

[31] Syed S, O’Sullivan TL, Phillips KP (2022). Extreme heat and pregnancy outcomes: a scoping review of the epidemiological evidence. Int J Environ Res Public Health.

[32] Richards M (2022). Acute association between heatwaves and stillbirth in six US states. Environ Health.

[33] McElroy S, Ilango S, Dimitrova A, Gershunov A, Benmarhnia T. Extreme heat, preterm birth, and stillbirth: a global analysis across 14 lower-middle income countries. Environ Int. 2022;158:106902. 10.1016/j.envint.2021.106902 34627013

[34] Chersich MF, et al. Associations between high temperatures in pregnancy and risk of preterm birth, low birth weight, and stillbirths: systematic review and meta-analysis. BMJ. 2020;371:m3811. 10.1136/bmj.m3811 PMC7610201 33148618

[35] Abubakar Kana M, Correia S, Peleteiro B, Severo M, Barros H (2017). Impact of the global financial crisis on low birth weight in Portugal: a time-trend analysis. BMJ Glob Health.

[36] Michas G, Varytimiadi A, Chasiotis I, Micha R (2014). Maternal and child mortality in Greece. Lancet.

[37] Margerison-Zilko ICE (2010). Economic contraction and birth outcomes: An integrative review. Hum Reprod Update.

[38] Stein Z, Susser M (1945). The Dutch Famine, 1944–1945, and the Reproductive Process. I. Effects Six Indices Birth.

[39] Brown AS, Susser ES (2008). Prenatal nutritional deficiency and risk of adult schizophrenia. Schizophrenia Bull.

[40] Butie C, Matthes KL, Hösli I, Floris J, Staub K (2020). Impact of World War 1 on placenta weight, birth weight and other anthropometric parameters of neonatal health. Placenta.

[41] Reid A (2005). The effects of the 1918–1919 influenza pandemic on infant and child health in Derbyshire. Med Hist.

[42] Almond D (2006). Is the 1918 Influenza Pandemic Over? Long-term effects of In utero influenza exposure in the post-1940 U.S. population. J Polit Econ.

[43] Lin M-J, Liu EM (2014). Does in utero exposure to Illness matter? The 1918 influenzaepidemic in Taiwan as a natural experiment. J Health Econ.

[44] Kotlar B, Gerson E, Petrillo S, Langer A, Tiemeier H (2021). The impact of the COVID-19 pandemic on maternal and perinatal health: a scoping review. Reprod Health.

[45] Hessami K, Romanelli C, Chiurazzi M, Cozzolino M (2022). COVID-19 pandemic and maternal mental health: a systematic review and meta-analysis. J Matern-Fetal Neonatal Med.

[46] Lambelet V (2021). Impact of the covid-19 pandemic on swiss pregnant and breastfeeding women - a cross-sectional study covering the first pandemic wave. Swiss Med Wkly.

[47] Yao XD, Li Y, Jiang H, Ma J, Wen J (2023). COVID-19 pandemic and neonatal birth weight: a systematic review and meta-analysis. Public Health.

[48] Blencowe H (2019). National, regional, and worldwide estimates of low birthweight in 2015, with trends from 2000: a systematic analysis. Lancet Glob Health.

[49] Federal Statistical Office. Infant mortality, stillbirths. 2022, https://www.bfs.admin.ch/bfs/en/home/statistiken/gesundheit/gesundheitszustand/sterblichkeit-todesursachen/saeuglings-totgeburten.html#accordion1685107995179.

[50] Skrivankova V. et al. Spatial epidemiology of gestational age and birth weight in Switzerland: Census-based linkage study. BMJ Open. 2019; 9. 10.1136/bmjopen-2018-027834 PMC6830696 31666260

[51] Matthes KL (2023). Long-term mortality effects of century crises: A warning from the past for the decades after COVID-19?. Swiss Med Wkly.

[52] Federal Statistical Office. Swiss official commune register. https://www.bfs.admin.ch/bfs/en/home/basics/swiss-official-commune-register.html.

[53] Federal Statistical Office. Structure de la population résidante permanente selon le canton, de 1999 à 2022. 2023. https://www.bfs.admin.ch/bfs/fr/home/statistiques/population/effectif-evolution.assetdetail.26565149.html.

[54] The Federal Assembly of the Swiss Confederation. Federal Act on Data Protection, 235.1. AS vol. 3387. 1992.

[55] Donoso E, Carvajal JA (2008). Maternal, perinatal and infant outcome of spontaneous pregnancy in the sixth decade of life. Maturitas.

[56] Bayrampour H, Heaman M (2010). Advanced maternal age and the risk of cesarean birth: a systematic review. Birth.

[57] Swiss Federal Statistical Office. Formular Gestationsalter. 2006.

[58] Panczak R, Berlin C, Voorpostel M, Zwahlen M, Egger M (2023). The Swiss neighbourhood index of socioeconomic position: update and re-validation. Swiss Med Wkly.

[59] Imfeld N et al. Hot and dry summers in Switzerlandd. Causes and impacts of the record summers 1947, 2003, and 2018. Geographica Bernensia. 2022. 10.4480/GB2022.G98.03.

[60] State Secretariat for Economic Affairs SECO. Gross domestic product quarterly data. 2023. https://www.seco.admin.ch/seco/en/home/wirtschaftslage---wirtschaftspolitik/Wirtschaftslage/bip-quartalsschaetzungen-/daten.html.

[61] Bundesamt für Gesundheit BAG, Bulletin. 20/10;20:535–43.

[62] Federal Office of Public Health. COVID-⁠19 Switzerland, Information on the current situation. https://www.covid19.admin.ch/en/epidemiologic/case?epiZoomDev=2020-02-24_2023-01-16.

[63] Delbaere I (2007). Should we adjust for gestational age when analysing birth weights? The use of z-scores revisited. Hum Reprod.

[64] Hernán MA, Hernández-Díaz S, Werler MM & Mitcheil AA. Causal knowledge as a prerequisite for confounding evaluation: an application to birth defects epidemiology. 2002; 155 https://academic.oup.com/aje/article/155/2/176/108106. 10.1093/aje/155.2.176 11790682

[65] Skrivankova VW. et al. Sociodemographic and regional differences in neonatal and infant mortality in Switzerland: The Swiss National Cohort. 2023. 10.1101/2023.09.19.23295765. 10.57187/s.3682 39835837

[66] Torche F (2011). The effect of maternal stress on birth outcomes: exploiting a natural experiment. Demography.

[67] Goin DE (2019). Exposure to community homicide during pregnancy and adverse birth outcomes: a within-community matched design. Epidemiology.

[68] Ellman LM (2008). Timing of fetal exposure to stress hormones: Effects on newborn physical and neuromuscular maturation. Dev Psychobiol.

[69] R Core Team. R: A language and environment for statistical computing. 2023. Preprint at https://www.R-project.org/.

[70] Wood SN (2017). General Addit Models.

[71] Yang H (2002). How does early ultrasound scan estimation of gestational age lead to higher rates of preterm birth?. Am J Obstet Gynecol.

[72] Modzelewska D, et al. Changes in data management contribute to temporal variation in gestational duration distribution in the Swedish Medical Birth Registry. PLoS One. 2020;15(11):e0241911. 10.1371/journal.pone.0241911 PMC7647076 33156833

[73] Swiss Federal Statistical Office. Reproductive health. https://www.bfs.admin.ch/bfs/en/home/statistics/health/state-health/reproductive-health.html.

[74] Betran AP, Torloni MR, Zhang JJ, Gülmezoglu AM (2016). WHO statement on caesarean section rates. BJOG.

[75] De Geyter C. Single embryo transfer in all infertile couples treated with assisted reproduction produces excellent results and avoids multiple births. Swiss Med Wkly. 2021;151:w20499. 10.4414/smw.2021.20499 34000057

[76] Schwarz C (2016). Temporal trends in fetal mortality at and beyond term and induction of labor in Germany 2005–2012: data from German routine perinatal monitoring. Arch Gynecol Obstet.

[77] Office fédéral de la statistique. Taux de chômeurs par canton. Valeurs mensuelles. 2023. https://www.bfs.admin.ch/bfs/fr/home/statistiques/travail-remuneration/chomage-sous-emploi/chomeurs-inscrits-seco.assetdetail.28405863.html.

[78] Yang J (2021). COVID-19 pandemic and population-level pregnancy and neonatal outcomes: a living systematic review and meta-analysis. Acta Obstetricia et Gynecologica Scandinavica.

[79] Calvert C (2023). Changes in preterm birth and stillbirth during COVID-19 lockdowns in 26 countries. Nat Hum Behav.

[80] Adams M, et al. Impact of SARS-CoV-2 on incidence, treatment and outcome of very preterm born infants in Switzerland: a retrospective, population-based cohort study. Swiss Med Wkly. 2022;152:w30174. 10.4414/smw.2022.w30174 35748336

[81] Dandona R, Kumar GA, Akbar M, Dora SSP, Dandona L. Substantial increase in stillbirth rate during the COVID-19 pandemic: Results from a population-based study in the Indian state of Bihar. BMJ Glob Health. 2023;8:e013021. 10.1136/bmjgh-2023-013021 PMC10373740 37491108

[82] Schwartz DA (2022). Placental Tissue Destruction and Insufficiency From COVID-19 Causes Stillbirth and Neonatal Death From Hypoxic-Ischemic Injury: A Study of 68 Cases With SARS-CoV-2 Placentitis From 12 Countries. Arch Pathol Lab Med.

[83] Khalil A (2020). Change in the Incidence of Stillbirth and Preterm Delivery during the COVID-19 Pandemic. JAMA.

[84] de Knegt VE, et al. The impact of the COVID-19 lockdown on birthweight among singleton term birth in Denmark. PLoS One. 2023;18(4):e0283909. 10.1371/journal.pone.0283909 PMC10118089 37079534

[85] Kotlar B, Gerson E, Petrillo S, Langer A, Tiemeier H. The impact of the COVID-19 pandemic on maternal and perinatal health: a scoping review. Reprod Health. 2021;18:10 (BioMed Central). 10.1186/s12978-021-01070-6 PMC7812564 33461593

[86] Allotey J, et al. Clinical manifestations, risk factors, and maternal and perinatal outcomes of coronavirus disease 2019 in pregnancy: living systematic review and meta-analysis. BMJ. 2020;370:m3320. 10.1136/bmj.m3320 PMC7459193 32873575

[87] Simbar, M., Nazarpour, S. & Sheidaei, A. Evaluation of pregnancy outcomes in mothers with COVID-19 infection: a systematic review and meta-analysis. J Obstet Gynaecol. 2023; 43. 10.1080/01443615.2022.2162867. 10.1080/01443615.2022.2162867 36651606

[88] Rytz R, Surbek D, Baud D & Ochsenbein N. Recommandation de La SSGO : Vaccination Contre Le COVID-19 Pendant La Grossesse et l’allaitement. 2021.

[89] Sobotka T (2023). Pandemic roller-coaster? Birth trends in higher-income countries during the COVID-19 pandemic. Popul Dev Rev.

[90] Flenady V. et al. Major risk factors for stillbirth in high-income countries: a systematic review and meta-analysis. 2011; 377. www.thelancet.com. 10.1016/S0140-6736(10)62233-7 21496916

[91] Shah PS (2010). Parity and low birth weight and preterm birth: a systematic review and meta-analyses. Acta Obstetricia et Gynecologica Scandinavica.

[92] Villadsen SF (2010). Cross-country variation in stillbirth and neonatal mortality in offspring of Turkish migrants in northern Europe. Eur J Public Health.

[93] de Sadovsky ADI, Mascarello KC, Miranda AE & Silveira MF. The associations that income, education, and ethnicity have with birthweight and prematurity: how close are they? Revista Panamericana de Salud Publica/Pan Am J Public Health. 2018; 42. 10.26633/RPSP.2018.92. 10.26633/RPSP.2018.92 PMC6385814 31093120

[94] Kierans WJ (2008). Does one size fit all? The case for ethnic-specific standards of fetal growth. BMC Pregnancy Childbirth.

[95] Buekens P, Masuy-Stroobant G & Delvaux T. High Birthweights among Infants of North African Immigrants in Belgium. 10.2105/ajph.88.5.808 PMC1508957 9585752

[96] Pereira PP da S, da Mata FAF, Figueiredo ACG, de Andrade KRC & Pereira MG. Maternal active smoking during pregnancy and low birth weight in the Americas: a systematic review and meta-analysis. Nicotine Tobacco Res. 2017; 19, 497–505. 10.1093/ntr/ntw228 28403455

[97] Federal Office of Public Health. COVID-19 Switzerland. 2023. https://www.covid19.admin.ch/en/epidemiologic/case/d/geo-regions?geoDate=2020-03-15.

[98] West EA (2020). Corona Immunitas: study protocol of a nationwide program of SARS-CoV-2 seroprevalence and seroepidemiologic studies in Switzerland. Int J Public Health.

[99] Zurich Canton. Daten, Proben & Datenschutz. https://www.zh.ch/de/gesundheit/ethik-humanforschung/daten-proben-datenschutz.html#-1296684915.

[100] Federal Statistical Office. Verordnung über die Durchführung von statistischen Erhebungen des Bundes, Art. 5. 1993. https://www.fedlex.admin.ch/eli/cc/1993/2100_2100_2100/de#art_5.

