# Supplementary material for: Health of singleton neonates in Switzerland through time and crises: a cross-sectional study at the population level, 2007-2022
Source: BMC Pregnancy Childbirth. 2024 Mar 25;24:218. doi: 10.1186/s12884-024-06414-1 (PMC10964517; doi:10.1186/s12884-024-06414-1)
Supplement: Supplementary file 1 — Supplementary Material 1. [file 12884_2024_6414_MOESM1_ESM.docx]

**Supplementary material**

**Figure S1:** Flowchart of exclusions


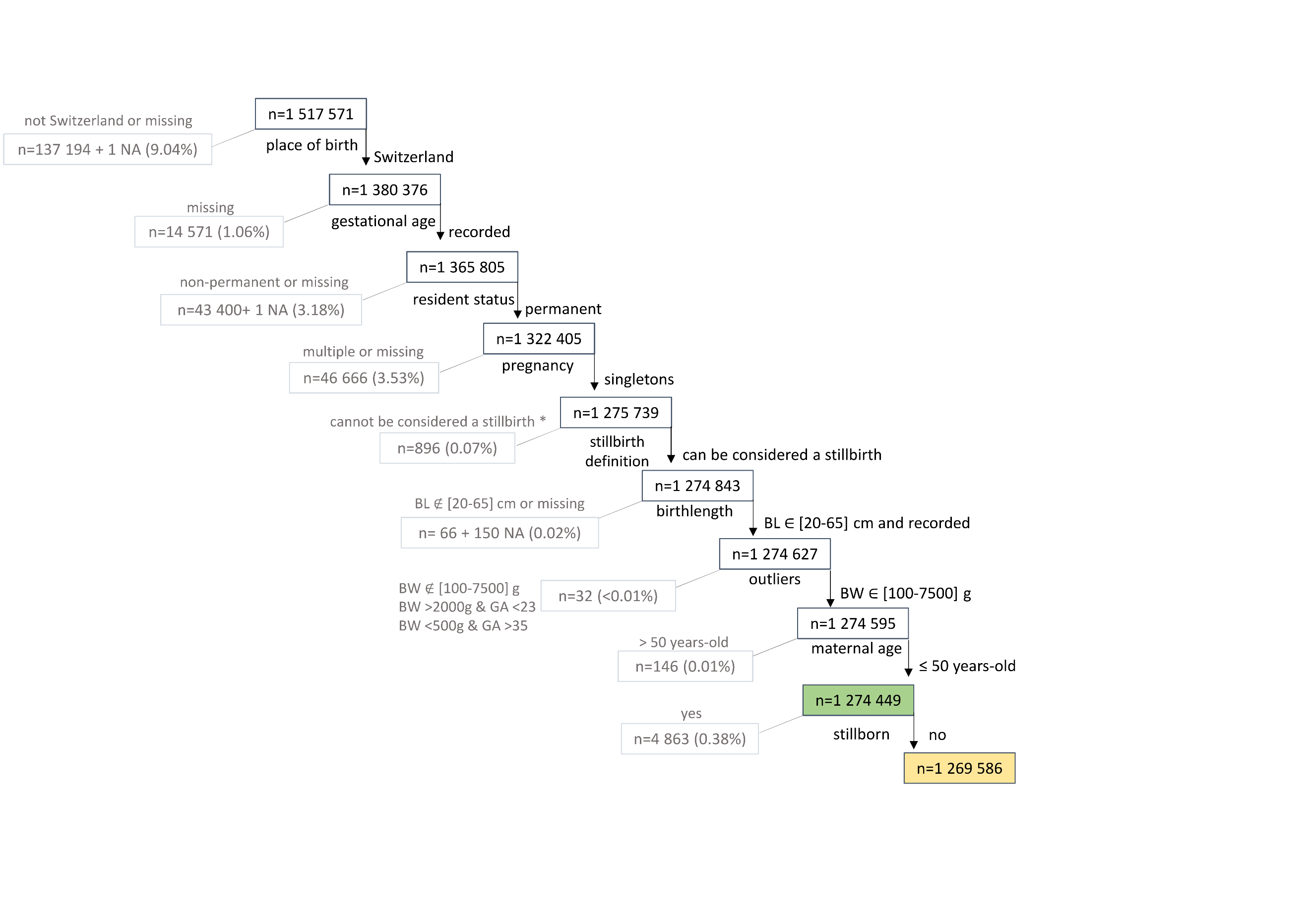


*: gestational age <22 weeks or birth weight <500 g (*n*=633), missing birth weight (*n*=263)

**Model parametrization:**

For all univariable models, the number of knots k was 10. The number of effective degrees of freedom (edf) was 7 for the univariable birth weight model and 1 for the PTB rate the stillbirth rate univariable models. In the multivariable models, maternal age was set with k=10 and edf=7, while seasonality was set with k=9 and edf=4. To asssess the fit and collinearity of our models, the *gam.check* and *concurvity* functions were respectively used. For the seasonality (birth month) smoothed term in the multivariable model, a cyclic spline was used, in order to account for the cyclical pattern of seasonality on pregnancy outcomes. The function *bam* from the *mgcv* package was used for all models.

**Equation E1:** Birth weight GAM, mathematical equation

$$Birth weight= \beta0 + \beta1 . time \left( numerical, going from month 1 to month 192 \right)+\beta2 . SEP \left( mean SSEP value of maternal municipality/10 points \right) +\beta3 . altitude \left( mean altitude of maternal municipality/100 MASL \right)+ \beta4 sex.I \left( neonatal sex is female \right) + \beta5 urbanity. I \left( maternal municipality is urban \right) + \beta6 civil status.I \left( civil status is single \right) + \sum_{k} \alpha k. I \left( individual i is of parity category k \right)+ \sum_{l} \gamma l. I \left( maternal municipality i is in language-region category l \right)+ \sum_{d} \delta d. I \left( maternal nationality i is of category d \right) +\beta7 heatwave.I \left( the mother was pregnant during a heatwave \right)+\beta8 greatrecession \left( continous relative exposure to the Great Recession during pregnancy \right)+\beta9 covid \left( continous relative exposure to COVID19 pandemic during pregnancy \right) +s maternal age \left( \mathrm{years} \right) +s seasonality \left( month of the year \right) +\varepsilon$$

With:

- β_0_ is an unknown coefficient corresponding to the intercept;
- β_1-3, 8-9_ are unknown coefficients corresponding to continuous variables;
- β_4-6, 7_ are unknown coefficients corresponding to binary variables;
- *α*k, *γ*l, *δ*d, are unknown coefficients corresponding to categorical variables with more than 2 levels;
- *I* is an indicator function (assumes a value of 1 if its argument is true, of 0 otherwise);
- *s* are smooth terms. *s_maternal age_* is implemented as thin plate regression splines ; *s_seasonality_* is implemented as a cyclic cubic spline;
- *ε* is an error term, that follows a gaussian distribution.

implemented as thin plate regression and cyclic cubic splines

**Equation E2:** GAM modelling PTB risk for exposure to crises during the first pregnancy trimester, R code

*m <- bam (preterm birth ~ s(maternal age) + s(month, bs=”cc”) + time (year and month of birth) + SSEP + altitude + parity + sex + urbanity + language region + maternal nationality + civil status + heatwave first trimester exposure + Great Recession first trimester exposure + COVID-19 first trimester exposure, family="binomial", data= population excl. stillbirths)*

**Table S1:** Maternal and neonatal characteristics of the eligible population

| **Birthyear** | **2007** | **2008** | **2009** | **2010** | **2011** | **2012** | **2013** | **2014** | **2015** | **2016** | **2017** | **2018** | **2019** | **2020** | **2021** | **2022** |
| --- | --- | --- | --- | --- | --- | --- | --- | --- | --- | --- | --- | --- | --- | --- | --- | --- |
| **Parity (%)** | | | | | | | | | | | | | | | | |
| 1 | 49.4 | 48.8 | 49.0 | 49.3 | 49.2 | 48.7 | 48.6 | 48.7 | 48.8 | 49.2 | 48.7 | 48.1 | 48.7 | 48.5 | 47.7 | 48.4 |
| 2 | 35.5 | 35.7 | 35.7 | 35.6 | 35.8 | 36.3 | 36.6 | 36.3 | 36.1 | 35.9 | 36.3 | 36.6 | 36.3 | 36.2 | 37.0 | 36.5 |
| 3 | 10.9 | 11.2 | 11.1 | 11.1 | 11.0 | 11.0 | 11.0 | 11.2 | 11.2 | 11.0 | 11.2 | 11.3 | 11.1 | 11.4 | 11.4 | 11.2 |
| >3 | 3.8 | 3.8 | 3.8 | 3.6 | 3.6 | 3.6 | 3.4 | 3.4 | 3.5 | 3.5 | 3.4 | 3.5 | 3.5 | 3.5 | 3.5 | 3.5 |
| missing | 0.4 | 0.4 | 0.4 | 0.4 | 0.4 | 0.4 | 0.4 | 0.4 | 0.4 | 0.4 | 0.4 | 0.4 | 0.4 | 0.3 | 0.4 | 0.4 |
| **Urbanity (%)** | | | | | | | | | | | | | | | | |
| Rural | 45.2 | 45.0 | 45.0 | 44.7 | 44.7 | 44.6 | 44.1 | 44.1 | 44.1 | 44.0 | 44.2 | 44.8 | 44.9 | 45.5 | 46.6 | 46.3 |
| Urban | 43.4 | 43.5 | 43.6 | 43.7 | 43.7 | 43.7 | 44.1 | 44.2 | 44.5 | 44.6 | 44.7 | 44.4 | 44.6 | 44.8 | 44.1 | 44.0 |
| missing | 11.4 | 11.5 | 11.4 | 11.6 | 11.6 | 11.7 | 11.8 | 11.7 | 11.4 | 11.4 | 11.1 | 10.8 | 10.4 | 9.8 | 9.3 | 9.7 |
| **Language region (%)** | | | | | | | | | | | | | | | | |
| German or Romansh | 61.8 | 62.1 | 62.0 | 62.0 | 62.3 | 62.5 | 62.3 | 62.5 | 63.0 | 62.9 | 63.2 | 63.7 | 63.9 | 64.6 | 65.1 | 64.2 |
| French | 23.3 | 22.8 | 23.2 | 23.0 | 22.8 | 22.6 | 22.8 | 22.7 | 22.5 | 22.8 | 22.8 | 22.9 | 22.9 | 22.9 | 22.9 | 23.3 |
| Italian | 3.4 | 3.6 | 3.4 | 3.4 | 3.4 | 3.2 | 3.2 | 3.1 | 3.1 | 3.0 | 2.9 | 2.7 | 2.7 | 2.7 | 2.7 | 2.8 |
| missing | 11.4 | 11.5 | 11.4 | 11.6 | 11.6 | 11.7 | 11.8 | 11.7 | 11.4 | 11.4 | 11.1 | 10.8 | 10.4 | 9.8 | 9.3 | 9.7 |
| **Maternal nationality (%)** | | | | | | | | | | | | | | | | |
| Switzerland | 61.6 | 61.4 | 61.2 | 60.4 | 60.4 | 59.7 | 59.2 | 58.6 | 58.5 | 57.7 | 57.8 | 58.2 | 58.2 | 59.0 | 59.5 | 58.7 |
| Africa | 2.5 | 2.5 | 2.6 | 2.5 | 2.8 | 2.9 | 3.1 | 3.0 | 3.2 | 3.5 | 3.6 | 3.5 | 3.5 | 3.3 | 3.1 | 3.3 |
| Asia | 3.8 | 3.6 | 3.5 | 3.4 | 3.5 | 3.7 | 3.7 | 3.8 | 3.9 | 4.4 | 4.2 | 4.2 | 4.1 | 4.0 | 3.9 | 4.0 |
| Europe | 28.2 | 28.1 | 28.2 | 29.2 | 29.1 | 29.5 | 30.0 | 30.5 | 30.5 | 30.7 | 30.8 | 30.7 | 31.1 | 30.7 | 30.7 | 31.2 |
| Northern America | 1.0 | 1.0 | 1.1 | 1.1 | 1.0 | 1.1 | 0.9 | 1.1 | 1.0 | 1.0 | 0.9 | 1.0 | 0.9 | 0.9 | 0.8 | 0.8 |
| Southern and Central America | 2.5 | 2.6 | 2.5 | 2.5 | 2.4 | 2.4 | 2.3 | 2.2 | 2.2 | 2.1 | 2.1 | 2.0 | 1.9 | 1.8 | 1.8 | 1.8 |
| Missing or Oceania | 0.4 | 0.8 | 1.1 | 0.8 | 0.7 | 0.8 | 0.9 | 0.7 | 0.7 | 0.7 | 0.5 | 0.5 | 0.3 | 0.3 | 0.3 | 0.2 |
| **Civil status (%)** | | | | | | | | | | | | | | | | |
| Married | 82.7 | 81.7 | 80.7 | 80.0 | 79.6 | 78.6 | 77.9 | 77.3 | 76.1 | 74.9 | 74.2 | 73.7 | 73.0 | 72.1 | 71.3 | 70.2 |
| Single | 17.3 | 18.3 | 19.3 | 20.0 | 20.4 | 21.4 | 22.1 | 22.7 | 23.9 | 25.1 | 25.8 | 26.3 | 27.0 | 27.9 | 28.7 | 29.8 |
| **Continuous variables** | | | | | | | | | | | | | | | | |
| Maternal age (years): mean ± sd | 30.9 ± 5.2 | 31 ± 5.2 | 31.1 ± 5.2 | 31.1 ± 5.2 | 31.3 ± 5.1 | 31.3 ± 5.1 | 31.4 ± 5.1 | 31.5 ± 5 | 31.6 ± 5 | 31.6 ± 5 | 31.7 ± 5 | 31.8 ± 4.9 | 31.9 ± 4.9 | 32 ± 4.8 | 32.1 ± 4.7 | 32.2 ± 4.8 |
| Mean SSEP: mean ± sd | 58.1 ± 8.7 | 58.2 ± 8.7 | 58.2 ± 8.7 | 58.2 ± 8.7 | 58.1 ± 8.6 | 58.1 ± 8.6 | 58.1 ± 8.6 | 58.2 ± 8.6 | 58.2 ± 8.6 | 58.1 ± 8.5 | 58.2 ± 8.6 | 58.2 ± 8.6 | 58.2 ± 8.5 | 58.1 ± 8.6 | 58.2 ± 8.6 | 58.1 ± 8.5 |
| Mean altitude (MASL): mean ± sd | 541 ± 192 | 541 ± 192 | 540 ± 191 | 538 ± 189 | 538 ± 188 | 539 ± 189 | 537 ± 188 | 537 ± 187 | 536 ± 186 | 536 ± 185 | 535 ± 184 | 536 ± 184 | 534 ± 182 | 534 ± 182 | 535 ± 183 | 536 ± 183 |
| **Neonatal outcomes** | | | | | | | | | | | | | | | | |
| Birth weight (g), mean ± sd | 3291 ± 562 | 3285 ± 568 | 3279 ± 570 | 3282 ± 571 | 3287 ± 568 | 3285 ± 573 | 3277 ± 569 | 3281 ± 566 | 3279 ± 566 | 3277 ± 567 | 3286 ± 562 | 3279 ± 567 | 3293 ± 555 | 3303 ± 556 | 3299 ± 557 | 3294 ± 560 |
| Gestational age (weeks), mean ± sd | 39.2 ± 2 | 39.1 ± 2.1 | 39.2 ± 2.1 | 39.2 ± 2.1 | 39.2 ± 2.1 | 39.2 ± 2.1 | 39.2 ± 2.1 | 39.2 ± 2.1 | 39.2 ± 2.1 | 39.2 ± 2.1 | 39.2 ± 2.1 | 39.2 ± 2.1 | 39.3 ± 2 | 39.3 ± 2 | 39.3 ± 2 | 39.3 ± 2.1 |
| Female (%) | 48.83 | 48.48 | 48.41 | 48.86 | 48.60 | 48.34 | 48.61 | 48.60 | 48.44 | 48.84 | 48.69 | 48.77 | 48.68 | 48.47 | 48.82 | 48.35 |
| Male (%) | 51.17 | 51.52 | 51.59 | 51.14 | 51.40 | 51.66 | 51.39 | 51.40 | 51.56 | 51.16 | 51.31 | 51.23 | 51.32 | 51.53 | 51.18 | 51.65 |
| Stillbirth (%) | 0.36 | 0.41 | 0.41 | 0.40 | 0.40 | 0.41 | 0.44 | 0.40 | 0.38 | 0.40 | 0.38 | 0.41 | 0.37 | 0.34 | 0.42 | 0.40 |
| Preterm birth (%) | 6.18 | 6.55 | 7.09 | 6.75 | 6.88 | 7.02 | 6.84 | 6.88 | 6.81 | 6.80 | 6.64 | 6.76 | 6.43 | 6.17 | 6.33 | 6.29 |
| Low birth weight (%) | 6.02 | 6.09 | 6.28 | 6.26 | 6.09 | 6.35 | 6.31 | 6.09 | 6.19 | 6.28 | 6.10 | 6.20 | 5.86 | 5.66 | 5.81 | 5.85 |
| n | 85893 | 88298 | 90097 | 92791 | 92324 | 94376 | 95010 | 97496 | 98688 | 100353 | 99050 | 99192 | 97042 | 95668 | 99479 | 91814 |

This dataset includes all births. sd: standard deviation. Total *n*= 1’517’571.

**Table S2:** maternal and neonatal characteristics of the analysed population (including stillbirths)

| **Birthyear** | **2007** | **2008** | **2009** | **2010** | **2011** | **2012** | **2013** | **2014** | **2015** | **2016** | **2017** | **2018** | **2019** | **2020** | **2021** | **2022** |
| --- | --- | --- | --- | --- | --- | --- | --- | --- | --- | --- | --- | --- | --- | --- | --- | --- |
| **Parity (%)** | | | | | | | | | | | | | | | | |
| 1 | 49.6 | 49.2 | 49.5 | 49.9 | 49.7 | 49.1 | 48.9 | 49.2 | 49.3 | 49.8 | 49.1 | 48.5 | 49.0 | 48.9 | 48.0 | 48.7 |
| 2 | 36.0 | 36.0 | 36.0 | 35.8 | 36.0 | 36.7 | 36.8 | 36.4 | 36.3 | 36.0 | 36.6 | 37.0 | 36.5 | 36.4 | 37.1 | 36.8 |
| 3 | 10.7 | 11.0 | 10.8 | 10.9 | 10.8 | 10.8 | 10.8 | 11.1 | 11.0 | 10.8 | 10.9 | 11.1 | 10.9 | 11.3 | 11.3 | 11.0 |
| >3 | 3.3 | 3.4 | 3.3 | 3.1 | 3.2 | 3.1 | 3.0 | 2.9 | 3.0 | 3.1 | 3.0 | 3.1 | 3.2 | 3.1 | 3.2 | 3.1 |
| missing | 0.3 | 0.4 | 0.4 | 0.4 | 0.4 | 0.4 | 0.4 | 0.4 | 0.4 | 0.4 | 0.4 | 0.4 | 0.4 | 0.3 | 0.4 | 0.4 |
| **Urbanity (%)** | | | | | | | | | | | | | | | | |
| Rural | 53.4 | 52.7 | 50.9 | 50.8 | 50.7 | 50.5 | 50.0 | 50.0 | 49.8 | 49.8 | 49.8 | 50.2 | 50.3 | 50.5 | 51.4 | 51.3 |
| Urban | 46.6 | 47.3 | 49.1 | 49.2 | 49.3 | 49.5 | 50.0 | 50.0 | 50.2 | 50.2 | 50.2 | 49.8 | 49.7 | 49.5 | 48.6 | 48.4 |
| missing | 0.0 | 0.0 | 0.0 | 0.0 | 0.0 | 0.0 | 0.0 | 0.0 | 0.0 | 0.0 | 0.0 | 0.0 | 0.0 | 0.0 | 0.0 | 0.3 |
| **Language region (%)** | | | | | | | | | | | | | | | | |
| German or Romansh | 76.5 | 75.1 | 70.0 | 70.1 | 70.5 | 70.8 | 70.7 | 70.8 | 71.1 | 71.0 | 71.2 | 71.5 | 71.3 | 71.6 | 71.8 | 71.0 |
| French | 19.1 | 20.5 | 26.1 | 26.0 | 25.7 | 25.6 | 25.8 | 25.6 | 25.4 | 25.6 | 25.6 | 25.5 | 25.6 | 25.3 | 25.2 | 25.7 |
| Italian | 4.4 | 4.4 | 3.9 | 3.8 | 3.8 | 3.6 | 3.6 | 3.6 | 3.5 | 3.4 | 3.3 | 3.0 | 3.0 | 3.1 | 3.0 | 3.1 |
| missing | 0.0 | 0.0 | 0.0 | 0.0 | 0.0 | 0.0 | 0.0 | 0.0 | 0.0 | 0.0 | 0.0 | 0.0 | 0.0 | 0.0 | 0.0 | 0.3 |
| **Maternal nationality (%)** | | | | | | | | | | | | | | | | |
| Switzerland | 65.1 | 64.7 | 63.6 | 62.9 | 62.4 | 61.7 | 61.3 | 60.6 | 60.2 | 59.5 | 59.6 | 60.0 | 60.1 | 60.6 | 61.2 | 60.7 |
| Africa | 1.7 | 1.8 | 2.2 | 2.1 | 2.7 | 2.8 | 3.1 | 2.9 | 3.2 | 3.5 | 3.7 | 3.5 | 3.5 | 3.3 | 3.1 | 3.2 |
| Asia | 3.4 | 3.1 | 3.0 | 3.0 | 3.1 | 3.3 | 3.2 | 3.4 | 3.5 | 4.0 | 3.9 | 3.9 | 3.8 | 3.9 | 3.7 | 3.8 |
| Europe | 27.2 | 27.4 | 27.7 | 28.7 | 28.7 | 28.9 | 29.2 | 30.0 | 30.1 | 30.2 | 30.1 | 30.0 | 30.3 | 30.0 | 29.8 | 30.0 |
| Northern America | 0.5 | 0.6 | 0.7 | 0.6 | 0.6 | 0.7 | 0.6 | 0.6 | 0.6 | 0.5 | 0.6 | 0.6 | 0.5 | 0.6 | 0.5 | 0.5 |
| Southern and Central America | 1.9 | 1.9 | 2.1 | 2.1 | 2.0 | 2.0 | 1.9 | 1.8 | 1.8 | 1.7 | 1.7 | 1.6 | 1.7 | 1.5 | 1.5 | 1.5 |
| Missing or Oceania | 0.2 | 0.5 | 0.7 | 0.5 | 0.5 | 0.6 | 0.7 | 0.6 | 0.6 | 0.6 | 0.4 | 0.4 | 0.2 | 0.2 | 0.2 | 0.2 |
| **Civil status (%)** | | | | | | | | | | | | | | | | |
| Married | 84.2 | 83.2 | 81.9 | 81.3 | 80.6 | 79.7 | 78.8 | 78.2 | 77.0 | 75.6 | 74.7 | 74.4 | 73.4 | 72.5 | 71.6 | 70.5 |
| Single | 15.8 | 16.8 | 18.1 | 18.7 | 19.4 | 20.3 | 21.2 | 21.8 | 23.0 | 24.4 | 25.3 | 25.6 | 26.6 | 27.5 | 28.4 | 29.5 |
| **Continuous variables** | | | | | | | | | | | | | | | | |
| Maternal age (years): mean ± sd | 30.8 ± 5.2 | 30.9 ± 5.2 | 31 ± 5.2 | 31 ± 5.1 | 31.1 ± 5.1 | 31.2 ± 5.1 | 31.2 ± 5 | 31.4 ± 5 | 31.4 ± 4.9 | 31.5 ± 5 | 31.6 ± 4.9 | 31.7 ± 4.9 | 31.8 ± 4.8 | 31.9 ± 4.8 | 32.1 ± 4.7 | 32.1 ± 4.7 |
| Mean SSEP: mean ± sd | 58.2 ± 8.6 | 58.2 ± 8.6 | 58.2 ± 8.7 | 58.2 ± 8.7 | 58.1 ± 8.6 | 58.1 ± 8.6 | 58.1 ± 8.6 | 58.1 ± 8.6 | 58.1 ± 8.6 | 58.1 ± 8.5 | 58.1 ± 8.6 | 58.2 ± 8.6 | 58.1 ± 8.5 | 58.1 ± 8.6 | 58.2 ± 8.6 | 58.1 ± 8.5 |
| Mean altitude (MASL): mean ± sd | 542 ± 190 | 541 ± 189 | 539 ± 189 | 538 ± 187 | 538 ± 188 | 538 ± 187 | 537 ± 186 | 537 ± 187 | 537 ± 186 | 536 ± 185 | 535 ± 183 | 536 ± 184 | 534 ± 182 | 534 ± 181 | 535 ± 182 | 536 ± 182 |
| **Neonatal outcomes** | | | | | | | | | | | | | | | | |
| Birth weight (g), mean ± sd | 3329 ± 523 | 3327 ± 527 | 3320 ± 526 | 3323 ± 528 | 3326 ± 526 | 3326 ± 531 | 3316 ± 529 | 3322 ± 522 | 3321 ± 522 | 3321 ± 520 | 3325 ± 521 | 3319 ± 523 | 3329 ± 516 | 3337 ± 518 | 3333 ± 519 | 3329 ± 520 |
| Gestational age (weeks), mean ± sd | 39.3 ± 1.8 | 39.3 ± 1.9 | 39.3 ± 1.9 | 39.4 ± 1.8 | 39.4 ± 1.8 | 39.3 ± 1.9 | 39.4 ± 1.9 | 39.4 ± 1.8 | 39.4 ± 1.8 | 39.4 ± 1.8 | 39.4 ± 1.8 | 39.4 ± 1.8 | 39.4 ± 1.8 | 39.4 ± 1.8 | 39.4 ± 1.8 | 39.4 ± 1.8 |
| Female (%) | 48.72 | 48.42 | 48.41 | 48.74 | 48.54 | 48.25 | 48.54 | 48.52 | 48.34 | 48.84 | 48.57 | 48.71 | 48.78 | 48.41 | 48.70 | 48.37 |
| Male (%) | 51.28 | 51.58 | 51.59 | 51.26 | 51.46 | 51.75 | 51.46 | 51.48 | 51.66 | 51.16 | 51.43 | 51.29 | 51.22 | 51.59 | 51.30 | 51.63 |
| Stillbirth (%) | 0.34 | 0.41 | 0.39 | 0.38 | 0.39 | 0.38 | 0.43 | 0.38 | 0.36 | 0.38 | 0.39 | 0.39 | 0.35 | 0.34 | 0.41 | 0.38 |
| Preterm birth (%) | 5.91 | 5.90 | 5.82 | 5.53 | 5.68 | 5.77 | 5.72 | 5.58 | 5.52 | 5.34 | 5.42 | 5.53 | 5.27 | 5.12 | 5.25 | 5.14 |
| Low birth weight (%) | 4.77 | 4.67 | 4.83 | 4.89 | 4.78 | 4.89 | 5.02 | 4.75 | 4.78 | 4.70 | 4.73 | 4.83 | 4.61 | 4.45 | 4.62 | 4.59 |
| **n** | 64785 | 69096 | 75268 | 77415 | 77903 | 79042 | 79649 | 82079 | 83352 | 84589 | 84075 | 84455 | 83241 | 83088 | 86810 | 79602 |

sd: standard deviation. Total *n*=1’274’449.

**Figure S2:** annual maternal and neonatal characteristics of the analysed population (including stillbirths): categorical variables


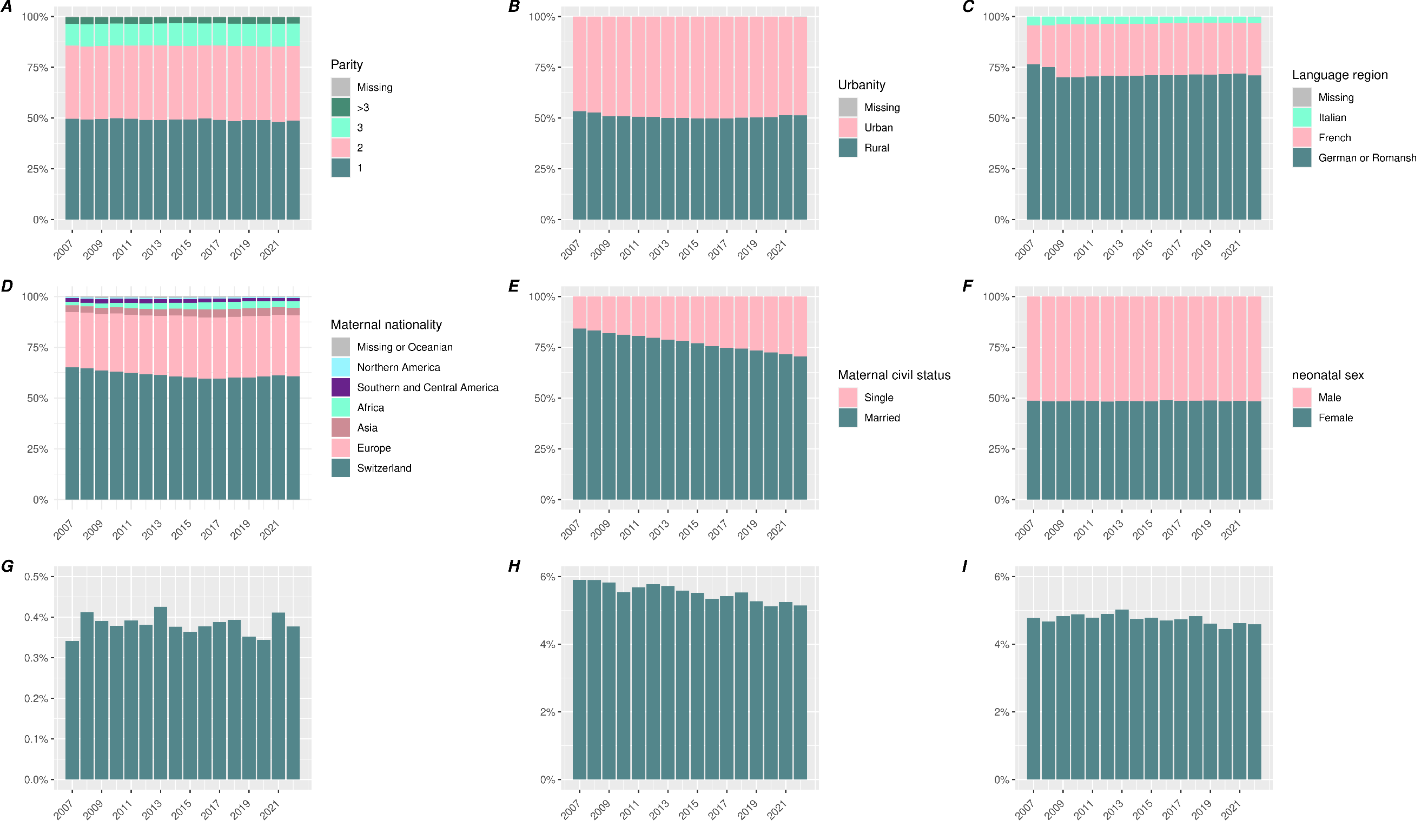


Parity (**A**), urbanity (**B**), language region (**C**), maternal nationality (**D**), civil status (**E**), neonatal sex (**F**), stillbirth (**G**), preterm birth (< 37 weeks, **H**), low birth weight (<2’500 g, **I**)

**Figure S3:** annual maternal and neonatal characteristics of the analysed population (including stillbirths): continuous variables


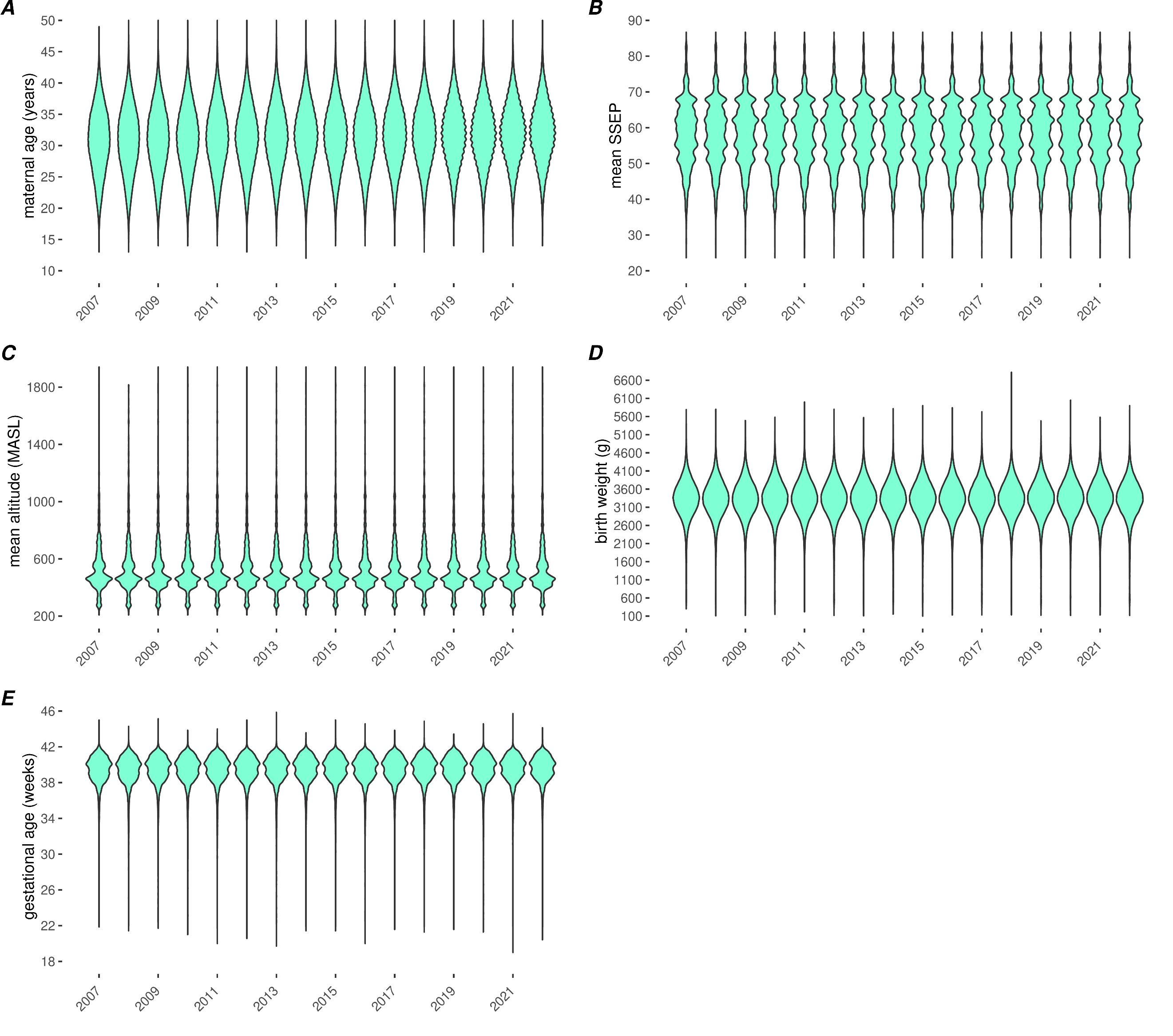


Maternal age (**A**), mean SSEP (**B**), mean altitude (**C**), birth weight (**D**), gestational age (**E**).

**Table S3:** maternal characteristics of the analysed population (excluding stillbirths)

| **Birthyear** | **2007** | **2008** | **2009** | **2010** | **2011** | **2012** | **2013** | **2014** | **2015** | **2016** | **2017** | **2018** | **2019** | **2020** | **2021** | **2022** |
| --- | --- | --- | --- | --- | --- | --- | --- | --- | --- | --- | --- | --- | --- | --- | --- | --- |
| **Parity (%)** | | | | | | | | | | | | | | | | |
| 1 | 49.8 | 49.4 | 49.7 | 50.1 | 49.8 | 49.3 | 49.2 | 49.4 | 49.5 | 50.0 | 49.3 | 48.7 | 49.2 | 49.1 | 48.2 | 48.8 |
| 2 | 36.1 | 36.2 | 36.1 | 35.9 | 36.1 | 36.8 | 37.0 | 36.6 | 36.4 | 36.1 | 36.7 | 37.1 | 36.6 | 36.5 | 37.3 | 37.0 |
| 3 | 10.7 | 11.1 | 10.9 | 10.9 | 10.8 | 10.8 | 10.8 | 11.1 | 11.1 | 10.9 | 11.0 | 11.1 | 11.0 | 11.3 | 11.3 | 11.1 |
| >3 | 3.3 | 3.4 | 3.3 | 3.1 | 3.2 | 3.1 | 3.0 | 2.9 | 3.0 | 3.1 | 3.0 | 3.1 | 3.2 | 3.2 | 3.2 | 3.1 |
| missing | 0 | 0 | 0 | 0 | 0 | 0 | 0 | 0 | 0 | 0 | 0 | 0 | 0 | 0 | 0 | 0 |
| **Urbanity (%)** | | | | | | | | | | | | | | | | |
| Rural | 53.4 | 52.7 | 50.9 | 50.8 | 50.7 | 50.5 | 50.1 | 50.0 | 49.9 | 49.8 | 49.8 | 50.2 | 50.3 | 50.5 | 51.4 | 51.3 |
| Urban | 46.6 | 47.3 | 49.1 | 49.2 | 49.3 | 49.5 | 49.9 | 50.0 | 50.1 | 50.2 | 50.2 | 49.8 | 49.7 | 49.5 | 48.6 | 48.4 |
| missing | 0.0 | 0.0 | 0.0 | 0.0 | 0.0 | 0.0 | 0.0 | 0.0 | 0.0 | 0.0 | 0.0 | 0.0 | 0.0 | 0.0 | 0.0 | 0.3 |
| **Language region (%)** | | | | | | | | | | | | | | | | |
| German or Romansh | 76.5 | 75.1 | 70.0 | 70.2 | 70.5 | 70.8 | 70.7 | 70.8 | 71.1 | 71.1 | 71.2 | 71.5 | 71.4 | 71.7 | 71.8 | 71.0 |
| French | 19.1 | 20.5 | 26.1 | 26.0 | 25.7 | 25.6 | 25.7 | 25.6 | 25.3 | 25.6 | 25.6 | 25.5 | 25.6 | 25.3 | 25.2 | 25.7 |
| Italian | 4.4 | 4.4 | 3.9 | 3.8 | 3.8 | 3.6 | 3.6 | 3.6 | 3.5 | 3.3 | 3.3 | 3.0 | 3.0 | 3.1 | 3.0 | 3.0 |
| missing | 0.0 | 0.0 | 0.0 | 0.0 | 0.0 | 0.0 | 0.0 | 0.0 | 0.0 | 0.0 | 0.0 | 0.0 | 0.0 | 0.0 | 0.0 | 0.3 |
| **Maternal nationality (%)** | | | | | | | | | | | | | | | | |
| Switzerland | 65.1 | 64.7 | 63.6 | 62.9 | 62.3 | 61.7 | 61.3 | 60.7 | 60.2 | 59.5 | 59.6 | 60.0 | 60.1 | 60.6 | 61.2 | 60.7 |
| Africa | 1.7 | 1.8 | 2.2 | 2.1 | 2.7 | 2.8 | 3.0 | 2.9 | 3.2 | 3.5 | 3.7 | 3.5 | 3.5 | 3.3 | 3.1 | 3.2 |
| Asia | 3.4 | 3.1 | 3.0 | 3.0 | 3.1 | 3.3 | 3.2 | 3.4 | 3.5 | 4.0 | 3.9 | 3.9 | 3.8 | 3.9 | 3.7 | 3.8 |
| Europe | 27.2 | 27.4 | 27.7 | 28.7 | 28.7 | 28.9 | 29.2 | 30.0 | 30.1 | 30.2 | 30.1 | 30.0 | 30.3 | 30.0 | 29.8 | 30.0 |
| Northern America | 0.5 | 0.6 | 0.7 | 0.6 | 0.6 | 0.7 | 0.6 | 0.6 | 0.6 | 0.5 | 0.6 | 0.6 | 0.5 | 0.6 | 0.5 | 0.6 |
| Southern and Central America | 1.9 | 1.9 | 2.1 | 2.1 | 2.0 | 2.0 | 1.9 | 1.8 | 1.8 | 1.7 | 1.7 | 1.6 | 1.7 | 1.5 | 1.5 | 1.5 |
| Missing or Oceania | 0.2 | 0.5 | 0.7 | 0.5 | 0.5 | 0.6 | 0.7 | 0.6 | 0.6 | 0.6 | 0.4 | 0.3 | 0.2 | 0.2 | 0.2 | 0.2 |
| **Civil status (%)** | | | | | | | | | | | | | | | | |
| Married | 84.2 | 83.3 | 82.0 | 81.3 | 80.6 | 79.7 | 78.8 | 78.3 | 77.0 | 75.6 | 74.7 | 74.4 | 73.5 | 72.5 | 71.6 | 70.5 |
| Single | 15.8 | 16.7 | 18.0 | 18.7 | 19.4 | 20.3 | 21.2 | 21.7 | 23.0 | 24.4 | 25.3 | 25.6 | 26.5 | 27.5 | 28.4 | 29.5 |
| **Continuous variables** | | | | | | | | | | | | | | | | |
| Maternal age (years): mean ± sd | 30.8 ± 5.2 | 30.9 ± 5.2 | 31 ± 5.2 | 31 ± 5.1 | 31.1 ± 5.1 | 31.2 ± 5.1 | 31.2 ± 5 | 31.4 ± 5 | 31.4 ± 4.9 | 31.5 ± 5 | 31.6 ± 4.9 | 31.7 ± 4.9 | 31.8 ± 4.8 | 31.9 ± 4.8 | 32.1 ± 4.7 | 32.1 ± 4.7 |
| Mean SSEP: mean ± sd | 58.2 ± 8.6 | 58.2 ± 8.6 | 58.2 ± 8.7 | 58.2 ± 8.7 | 58.1 ± 8.6 | 58.1 ± 8.6 | 58.1 ± 8.6 | 58.1 ± 8.6 | 58.1 ± 8.6 | 58.1 ± 8.5 | 58.1 ± 8.6 | 58.2 ± 8.6 | 58.1 ± 8.5 | 58.1 ± 8.6 | 58.2 ± 8.6 | 58.1 ± 8.5 |
| Mean altitude (MASL): mean ± sd | 542 ± 190 | 541 ± 189 | 539 ± 189 | 538 ± 187 | 538 ± 188 | 538 ± 187 | 537 ± 186 | 537 ± 187 | 537 ± 186 | 536 ± 185 | 535 ± 183 | 536 ± 184 | 534 ± 182 | 534 ± 181 | 535 ± 182 | 536 ± 182 |
| **Neonatal outcomes** | | | | | | | | | | | | | | | | |
| Birth weight (g), mean ± sd | 3334 ± 511 | 3334 ± 511 | 3327 ± 510 | 3330 ± 511 | 3333 ± 511 | 3333 ± 514 | 3324 ± 510 | 3328 ± 508 | 3328 ± 507 | 3327 ± 505 | 3333 ± 505 | 3326 ± 507 | 3335 ± 502 | 3343 ± 505 | 3340 ± 503 | 3336 ± 503 |
| Gestational age (weeks), mean ± sd | 39.3 ± 1.7 | 39.3 ± 1.7 | 39.4 ± 1.7 | 39.4 ± 1.7 | 39.4 ± 1.7 | 39.4 ± 1.7 | 39.4 ± 1.7 | 39.4 ± 1.7 | 39.4 ± 1.7 | 39.4 ± 1.7 | 39.4 ± 1.7 | 39.4 ± 1.7 | 39.4 ± 1.7 | 39.4 ± 1.7 | 39.4 ± 1.7 | 39.4 ± 1.7 |
| Female (%) | 48.72 | 48.43 | 48.39 | 48.72 | 48.52 | 48.27 | 48.53 | 48.50 | 48.36 | 48.84 | 48.57 | 48.71 | 48.78 | 48.41 | 48.69 | 48.37 |
| Male (%) | 51.28 | 51.57 | 51.61 | 51.28 | 51.48 | 51.73 | 51.47 | 51.50 | 51.64 | 51.16 | 51.43 | 51.29 | 51.22 | 51.59 | 51.31 | 51.63 |
| Preterm birth (%) | 5.69 | 5.62 | 5.54 | 5.26 | 5.41 | 5.49 | 5.42 | 5.34 | 5.26 | 5.09 | 5.13 | 5.24 | 5.03 | 4.88 | 4.96 | 4.87 |
| Low birth weight (%) | 4.55 | 4.39 | 4.56 | 4.61 | 4.51 | 4.62 | 4.70 | 4.49 | 4.52 | 4.44 | 4.45 | 4.53 | 4.37 | 4.21 | 4.33 | 4.31 |
| **n** | 64564 | 68811 | 74974 | 77122 | 77598 | 78741 | 79310 | 81770 | 83049 | 84270 | 83749 | 84123 | 82948 | 82802 | 86453 | 79302 |

sd: standard deviation. Total *n*= 1’269’586.

**Table S4:** birth rate among permanent residents (whole eligible population)

| **Birthyear** | **2007** | **2008** | **2009** | **2010** | **2011** | **2012** | **2013** | **2014** | **2015** | **2016** | **2017** | **2018** | **2019** | **2020** | **2021** | **2022** |
| --- | --- | --- | --- | --- | --- | --- | --- | --- | --- | --- | --- | --- | --- | --- | --- | --- |
| n | 74834 | 77088 | 78636 | 80825 | 81141 | 82652 | 83097 | 85608 | 86971 | 88423 | 87647 | 88113 | 86516 | 86121 | 89874 | 82501 |
| Birth rate (/1000 inhabitants) | 9.86 | 10.01 | 10.10 | 10.27 | 10.20 | 10.28 | 10.21 | 10.39 | 10.44 | 10.50 | 10.33 | 10.31 | 10.05 | 9.93 | 10.28 | 9.36 |

**Figure S4:** Monthly birth rate among the permanent resident population


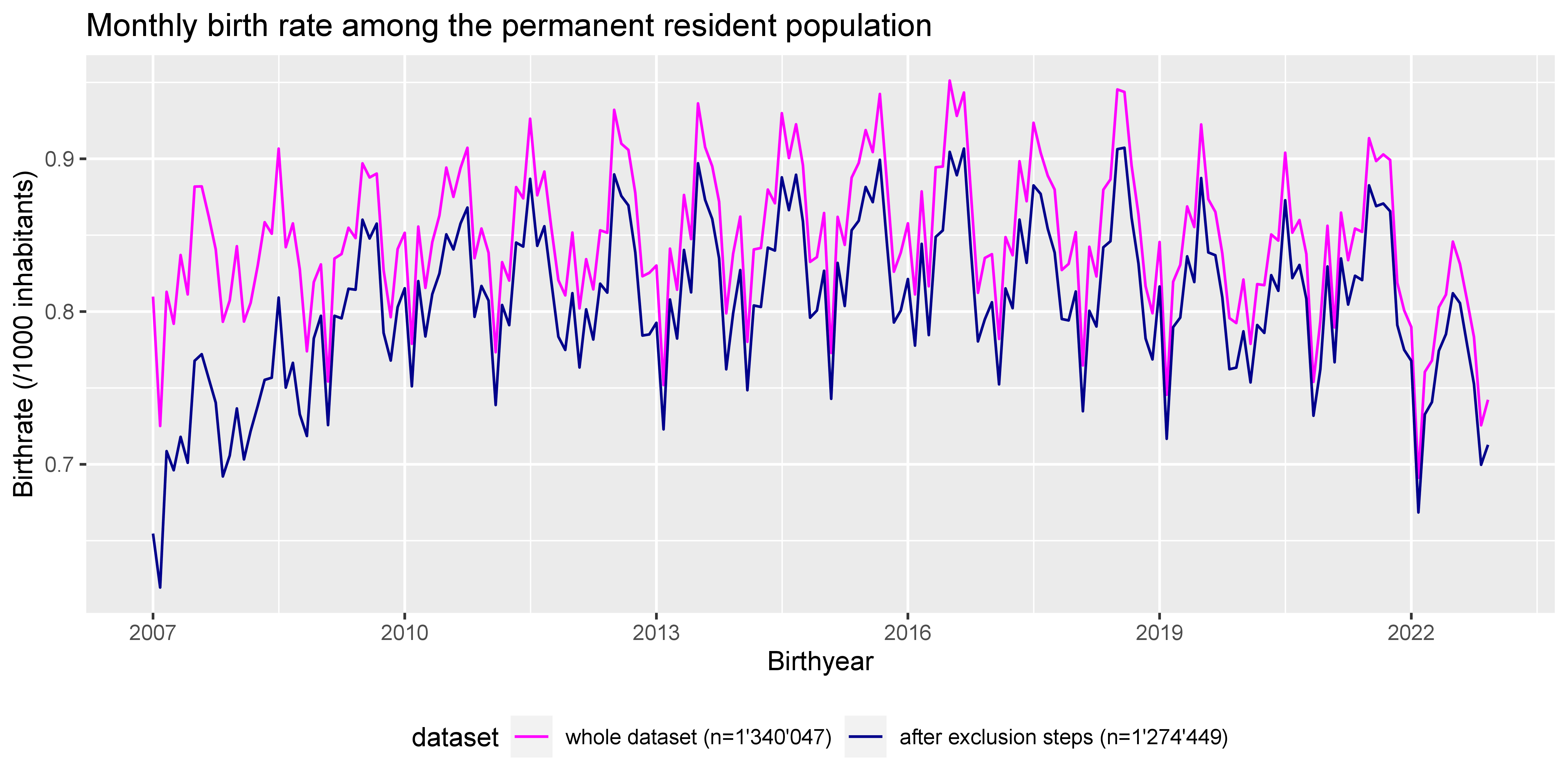


This is among the whole eligible population and analysed population (incl. stillbirth). Note: the gap between the two plots in 2007-2008

is due to the fact that data where gestational age was not recorded was excluded (blue line), and gestational age was missing frequently

2007-2008.

**Figure S5**: Birth weight GAM: smooth variables (model 1.1).**
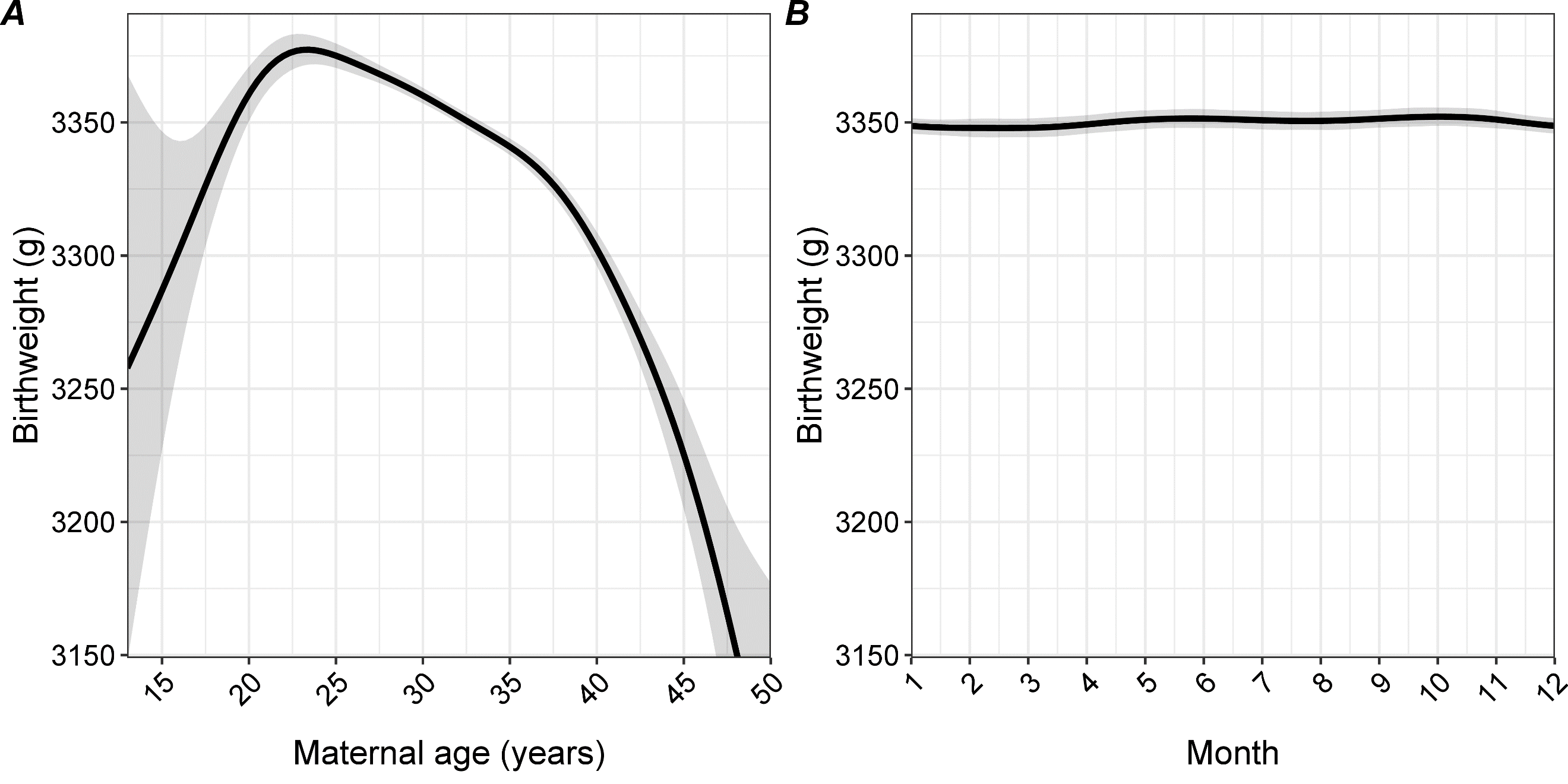
**

This corresponds to Table 2 in the main text. **A**: maternal age. **B:** Seasonality**.**

**Figure S6:** Preterm birth GAM: smooth variables (model 2.1).**
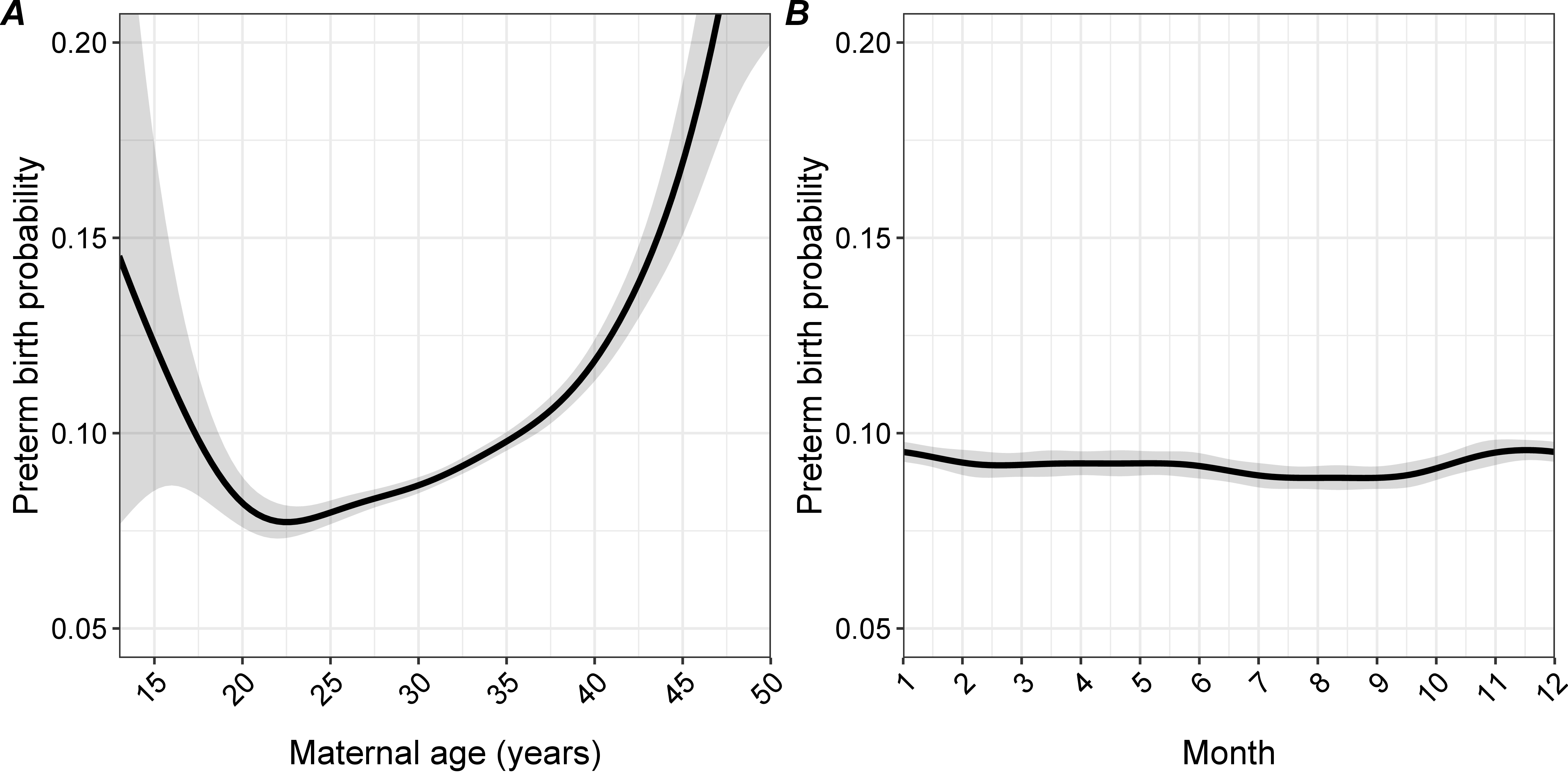
**

This corresponds to Table 3 in the main text. **A**: maternal age. **B:** Seasonality**.**

**Figure S7:** Stillbirth GAM: smooth variables (model 3.1).**
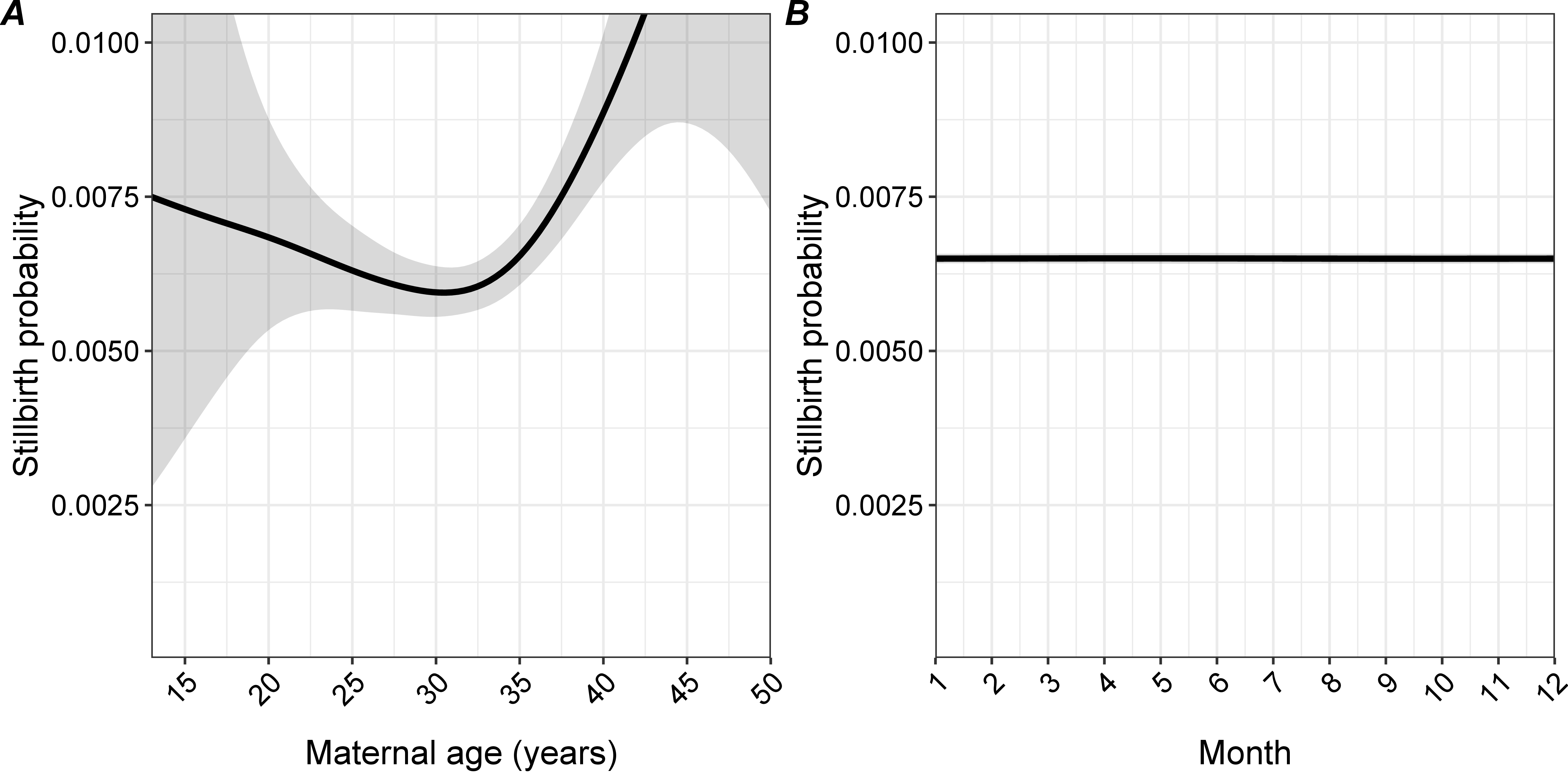
**

This corresponds to Table 3 in the main text. **A**: maternal age. **B:** Seasonality**.**

**Flu exposure**

**Table S5:** Birth weight linear regression GAM with flu exposure variable (model 1.2).

|  |  |  | **95% CI (g)** | |  |
| --- | --- | --- | --- | --- | --- |
| **Variable** | **Category** | **beta** | **lci** | **uci** | **d** |
| Time (by month) |  | 0.05 | 0.02 | 0.07 | 0.00 |
| SSEP (/10 points) |  | 4.55 | 3.42 | 5.67 | 0.01 |
| Altitude (/100m) |  | -6.99 | -7.49 | -6.48 | 0.02 |
| Parity (ref: 1) | 2 | 123.41 | 121.46 | 125.36 | 0.11 |
|  | 3 | 163.35 | 160.37 | 166.33 | 0.10 |
|  | >3 | 188.44 | 183.28 | 193.60 | 0.06 |
| Sex (ref: male) | Female | -132.26 | -133.99 | -130.52 | 0.13 |
| Urban (ref: rural) | Urban | 2.07 | 0.25 | 3.89 | 0.00 |
| Language region (ref: German) | French | -53.02 | -55.13 | -50.91 | 0.04 |
|  | Italian | -89.20 | -94.06 | -84.33 | 0.03 |
| Maternal nationality (ref: Swiss) | Africa | 11.92 | 6.67 | 17.17 | 0.00 |
|  | Asia | -58.82 | -63.59 | -54.04 | 0.02 |
|  | Europe | 27.10 | 25.12 | 29.08 | 0.02 |
|  | Northern America | 71.72 | 60.35 | 83.09 | 0.01 |
|  | Southern/Central America | 5.58 | -0.99 | 12.16 | 0.00 |
| Civil status (ref: married) | Single | -37.88 | -40.03 | -35.74 | 0.03 |
| Heatwave (ref: 0) | 1 | 9.00 | 6.00 | 12.01 | 0.01 |
| Flu pandemic (continuous) |  | 2.27 | -5.73 | 10.28 | 0.00 |
| COVID (continuous) |  | 10.71 | 4.54 | 16.88 | 0.00 |
| **Smooth variables** |  |  |  |  | **p-value** |
| Maternal age (years) |  |  |  |  | <0.0001 |
| Seasonality (month) |  |  |  |  | <0.01 |
| *n*=1’263’853 | | | | | |

#### 95%CI: 95% Confidence interal, lci: lower confidence interval, uci: upper confidence interval, d: Cohen’s d.

#### Cohen’s d >0.1, >0.3, >0.5 are respectively considered small, moderate and large effect sizes. Great Recession

#### and COVID-19 exposure variables are relative to pregnancy duration (values between 0 and 1). SSEP scale goes

#### from 23.6 to 86.7, by 10 points increase.

**Figure S8:** Birth weight linear regression GAM with flu exposure variable (model 1.2), smooth variables.

**
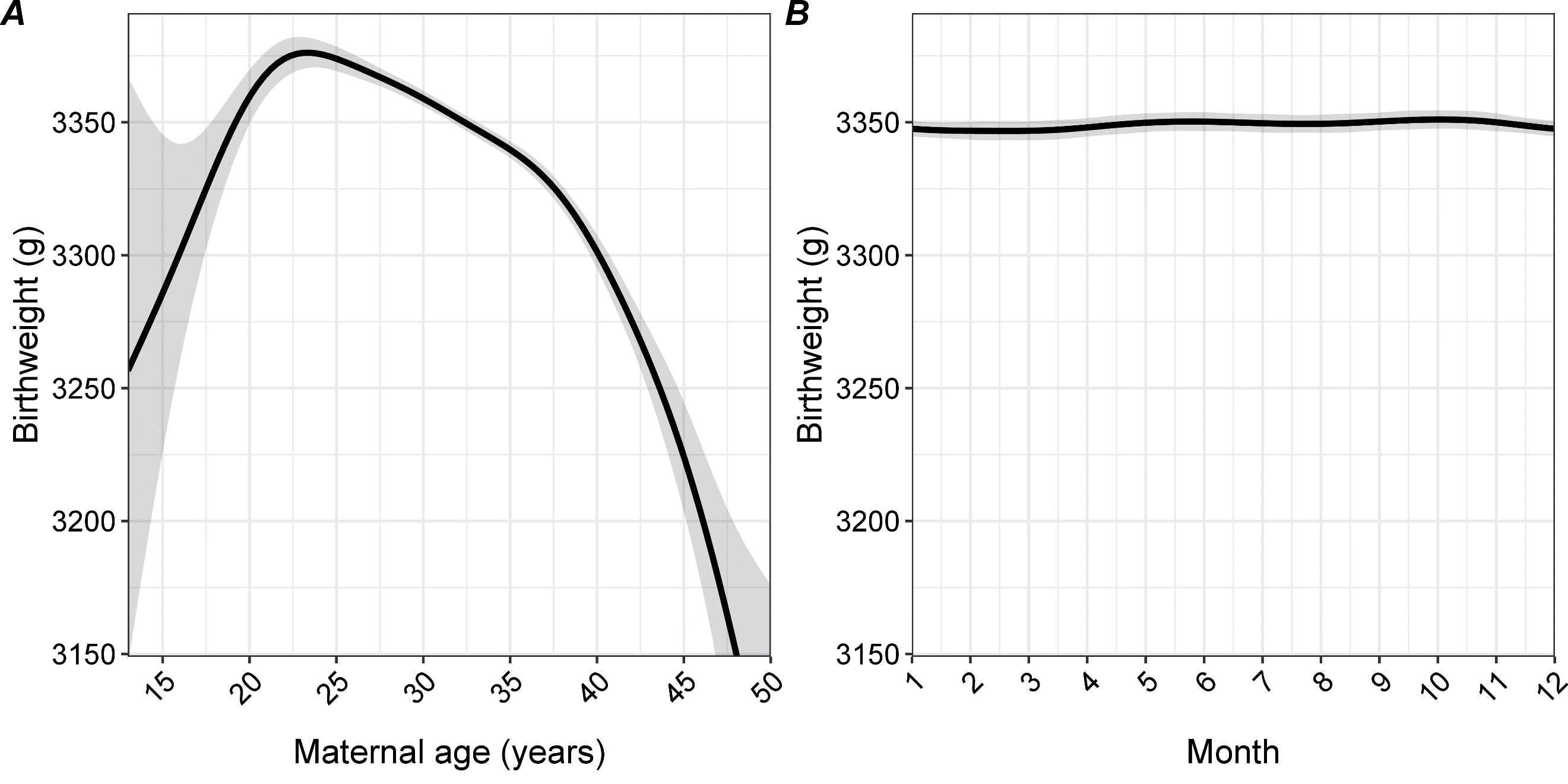
**

**A**: maternal age. **B:** Seasonality**.**

**Table S6:** Preterm birth logistic regression GAM with flu exposure variable (model 2.2).

|  |  |  | **95% CI** | |  |
| --- | --- | --- | --- | --- | --- |
| **Variable** | **Category** | **OR** | **lci** | **uci** | **d** |
| Time (by month) |  | 1.00 | 1.00 | 1.00 | 0.00 |
| SSEP (/10 points) |  | 0.96 | 0.95 | 0.97 | 0.02 |
| Altitude (/100m) |  | 1.00 | 1.00 | 1.00 | 0.00 |
| Parity (ref: 1) | 2 | 0.67 | 0.66 | 0.69 | 0.22 |
|  | 3 | 0.68 | 0.67 | 0.70 | 0.21 |
|  | >3 | 0.78 | 0.74 | 0.81 | 0.14 |
| Sex (ref: male) | Female | 0.84 | 0.83 | 0.85 | 0.10 |
| Urban (ref: rural) | Urban | 0.95 | 0.94 | 0.97 | 0.03 |
| Language region (ref: German) | French | 1.05 | 1.03 | 1.07 | 0.03 |
|  | Italian | 1.00 | 0.96 | 1.05 | 0.00 |
| Maternal nationality (ref: Swiss) | Africa | 1.00 | 0.96 | 1.05 | 0.00 |
|  | Asia | 1.07 | 1.03 | 1.12 | 0.04 |
|  | Europe | 0.95 | 0.93 | 0.97 | 0.03 |
|  | Northern America | 0.85 | 0.76 | 0.94 | 0.09 |
|  | Southern/Central America | 1.09 | 1.03 | 1.15 | 0.05 |
| Civil status (ref: married) | Single | 1.12 | 1.10 | 1.14 | 0.06 |
| Heatwave (ref: 0) | 1 | 0.85 | 0.82 | 0.87 | 0.09 |
| Flu pandemic (continuous) |  | 0.89 | 0.83 | 0.95 | 0.07 |
| COVID (continuous) |  | 0.99 | 0.93 | 1.04 | 0.01 |
| **Smooth variables** |  |  |  |  | **p-value** |
| Maternal age (years) |  |  |  |  | <0.0001 |
| Seasonality (month) |  |  |  |  | <0.0001 |
| *n*=1,263,853 | | | | | |

95%CI: 95% Confidence interal, lci: lower confidence interval, uci: upper confidence interval, d:

Cohen’s d. Cohen’s d >0.1, >0.3, >0.5 are respectively considered small, moderate and large effect sizes.

#### Great Recession and COVID-19 exposure variables are relative to pregnancy duration (values between 0

#### and 1). SSEP scale goes from 23.6 to 86.7, by 10 points increase.

**Figure S9**: Preterm birth logistic regression GAM with flu exposure variable (model 2.2), smooth variables.


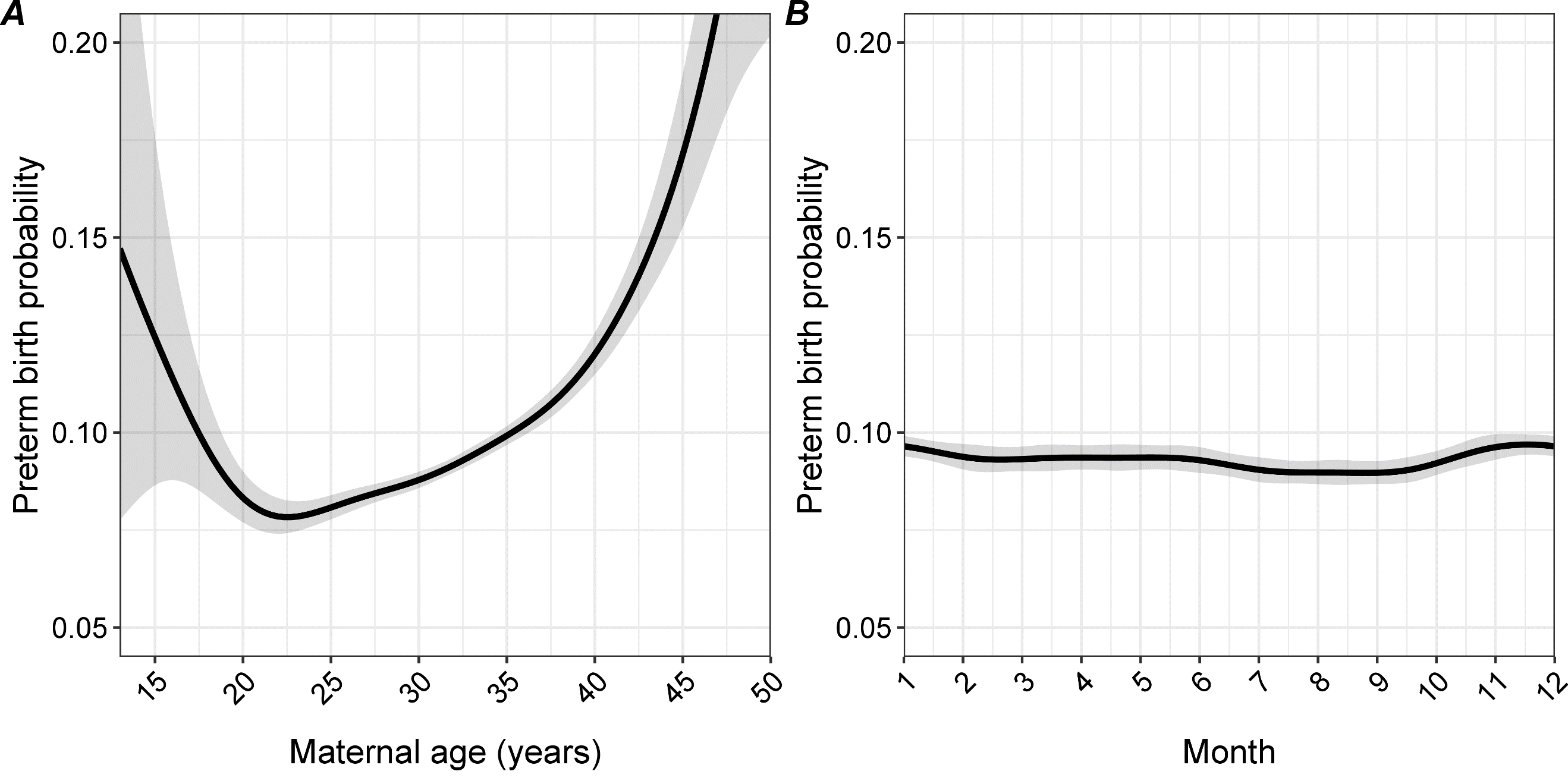


**A**: maternal age. **B**: Seasonality.

**Table S7:** Stillbirth logistic regression GAM with flu exposure variable (model 3.2).

|  |  |  | **95% CI** | |  |
| --- | --- | --- | --- | --- | --- |
| **Variable** | **Category** | **OR** | **lci** | **uci** | **d** |
| Time (by month) |  | 1.00 | 1.00 | 1.00 | 0.00 |
| SSEP (/10 points) |  | 0.90 | 0.87 | 0.93 | 0.06 |
| Altitude (/100m) |  | 0.99 | 0.97 | 1.00 | 0.01 |
| Sex (ref: male) | Female | 1.01 | 0.95 | 1.07 | 0.01 |
| Urban (ref: rural) | Urban | 1.06 | 0.99 | 1.12 | 0.03 |
| Language region (ref: German) | French | 1.14 | 1.06 | 1.22 | 0.07 |
|  | Italian | 0.94 | 0.80 | 1.10 | 0.03 |
| Maternal nationality (ref: Swiss) | Not Swiss | 1.13 | 1.06 | 1.19 | 0.07 |
| Civil status (ref: married) | Single | 1.38 | 1.29 | 1.47 | 0.18 |
| Heatwave (ref: 0) | 1 | 0.77 | 0.69 | 0.86 | 0.14 |
| Flu pandemic (continuous) |  | 1.09 | 0.85 | 1.41 | 0.05 |
| COVID (continuous) |  | 1.15 | 0.94 | 1.40 | 0.08 |
| **Smooth variables** |  |  |  |  | **p-value** |
| Maternal age (years) |  |  |  |  | <0.0001 |
| Seasonality (month) |  |  |  |  | 0.70 |
| *n*=1’270’114 | | | | | |

95%CI: 95% Confidence interval, OR: Odds-ratio, lci: lower confidence interval,

#### uci: upper confidence interval, d: Cohen’s d. Cohen’s d >0.1, >0.3, >0.5 are

#### respectively considered small, moderate and large effect sizes. Great

#### Recession and COVID-19 exposure variables are relative to pregnancy duration

#### (values between 0 and 1). SSEP scale goes from 23.6 to 86.7, by 10 points increase.

**Figure S10**: Stillbirth logistic regression GAM with flu exposure variable (model 3.2), smooth variables.


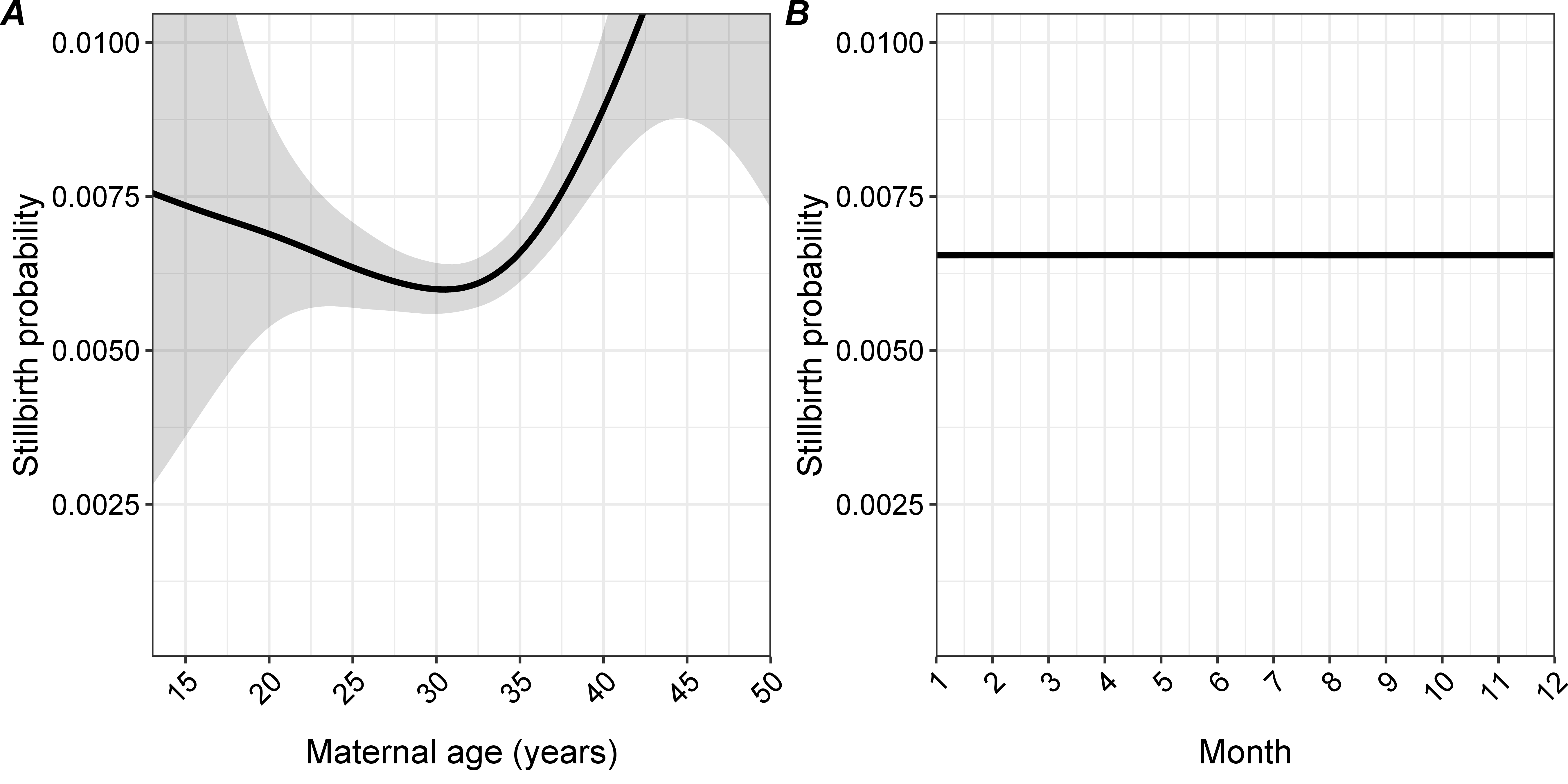


**A**: maternal age. **B**: Seasonality.

**Table S8**: trimester effect of crises on birth weight, preterm birth and stillbirth.

|  |  | **Birth weight** | | | | **Preterm birth** | | | | **Stillbirth** | | | |
| --- | --- | --- | --- | --- | --- | --- | --- | --- | --- | --- | --- | --- | --- |
| **Crisis** | **Trimester** | **beta (g)** | **lci (g)** | **uci (g)** | **d** | **OR** | **lci** | **uci** | **d** | **OR** | **lci** | **uci** | **d** |
| **Heatwave** | **First** | 0.20 | -4.92 | 5.31 | 0.00 | 0.95 | 0.91 | 1.00 | 0.03 | 0.97 | 0.82 | 1.15 | 0.02 |
| **Flu** |  | 1.43 | -5.68 | 8.53 | 0.00 | 0.94 | 0.89 | 1.01 | 0.03 | 1.05 | 0.84 | 1.32 | 0.03 |
| **COVID-19** |  | -6.27 | -12.17 | -0.37 | 0.00 | 1.06 | 1.00 | 1.12 | 0.03 | 1.04 | 0.86 | 1.26 | 0.02 |
| **Heatwave** | **Last** | -10.92 | -15.85 | -5.99 | 0.00 | 1.03 | 0.98 | 1.07 | 0.01 | 0.99 | 0.85 | 1.16 | 0.00 |
| **Flu** |  | 4.96 | -2.43 | 12.35 | 0.00 | 0.93 | 0.87 | 1.00 | 0.04 | 0.93 | 0.73 | 1.19 | 0.04 |
| **COVID-19** |  | 16.62 | 10.47 | 22.77 | 0.00 | 0.98 | 0.93 | 1.04 | 0.01 | 1.24 | 1.03 | 1.51 | 0.12 |

OR: Odds-ratio, lci: lower confidence interval, uci: upper confidence interval, d: Cohen’s d. Corresponding models are

model 1.2.A and 1.2.B (birth weight), model 2.2.A and 2.2.B (preterm birth), and (model 3.2.A and 3.2.B stillbirth). Cohen’s

d >0.1, >0.3, >0.5 are respectively considered small, moderate and large effect sizes.

**Sensitivity analysis with low birth weight (birth weight < 2’500g):**

#### **Figure S11:** Association between low birth weight rate and birthdate from a GAM.


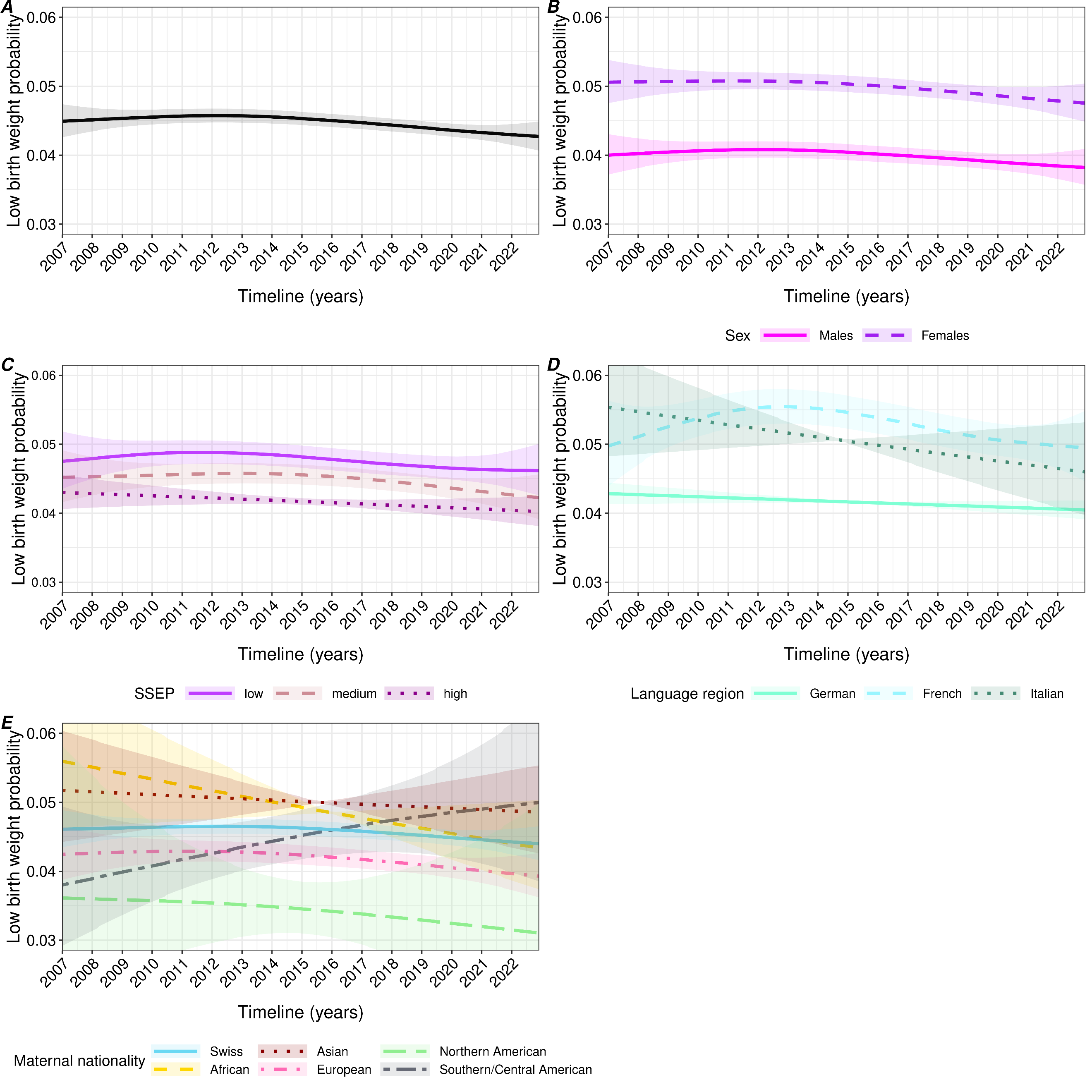


All models are univariable. **A**: unstratified. The other graphs are stratified by: sex (**B**), SSEP (**C**), language-region (**D**), and maternal nationality (**E**).

**Table S9:** Low birth weight logistic regression GAM (model 4.1).

|  |  |  | **95% CI** | |  |
| --- | --- | --- | --- | --- | --- |
| **Variable** | **Category** | **OR** | **lci** | **uci** | **d** |
| Heatwave (ref: 0) | 1 | 0.89 | 0.87 | 0.92 | 0.06 |
| Great Recession (continuous) |  | 1.02 | 0.94 | 1.11 | 0.01 |
| COVID (continuous) |  | 0.96 | 0.90 | 1.02 | 0.03 |
| Time (by month) |  | 1.00 | 1.00 | 1.00 | 0.00 |
| SSEP (/10 points) |  | 0.93 | 0.92 | 0.94 | 0.04 |
| Altitude (/100m) |  | 1.02 | 1.02 | 1.03 | 0.01 |
| Parity (ref: 1) | 2 | 0.54 | 0.53 | 0.55 | 0.34 |
|  | 3 | 0.52 | 0.50 | 0.54 | 0.36 |
|  | >3 | 0.56 | 0.53 | 0.59 | 0.32 |
| Sex (ref: male) | Female | 1.26 | 1.24 | 1.28 | 0.13 |
| Urban (ref: rural) | Urban | 0.98 | 0.96 | 1.00 | 0.01 |
| Language region (ref: German) | French | 1.20 | 1.18 | 1.23 | 0.10 |
|  | Italian | 1.15 | 1.10 | 1.21 | 0.08 |
| Maternal nationality (ref: Swiss) | Africa | 1.07 | 1.02 | 1.12 | 0.04 |
|  | Asia | 1.15 | 1.10 | 1.20 | 0.08 |
|  | Europe | 0.89 | 0.88 | 0.91 | 0.06 |
|  | Northern America | 0.69 | 0.61 | 0.78 | 0.21 |
|  | Southern/Central America | 0.91 | 0.86 | 0.97 | 0.05 |
| Civil status (ref: married) | Single | 1.25 | 1.23 | 1.28 | 0.13 |
| **Smooth variables** |  |  |  |  | **p-value** |
| Maternal age (years) |  |  |  |  | <0.0001 |
| Seasonality (month) |  |  |  |  | <0.001 |
| n=1’263’853 |  |  |  |  |  |

95%CI: 95% Confidence interal, lci: lower confidence interval, uci: upper confidence interval,

d: Cohen’s d. Cohen’s d >0.1, >0.3, >0.5 are respectively considered small, moderate and

large effect sizes. Great Recession and COVID-19 exposure variables are relative to pregnancy

duration (values between 0 and 1). SSEP scale goes from 23.6 to 86.7, by 10 points increase.

**Figure S12:** Low birth weight logistic regression GAM (model 4.1).


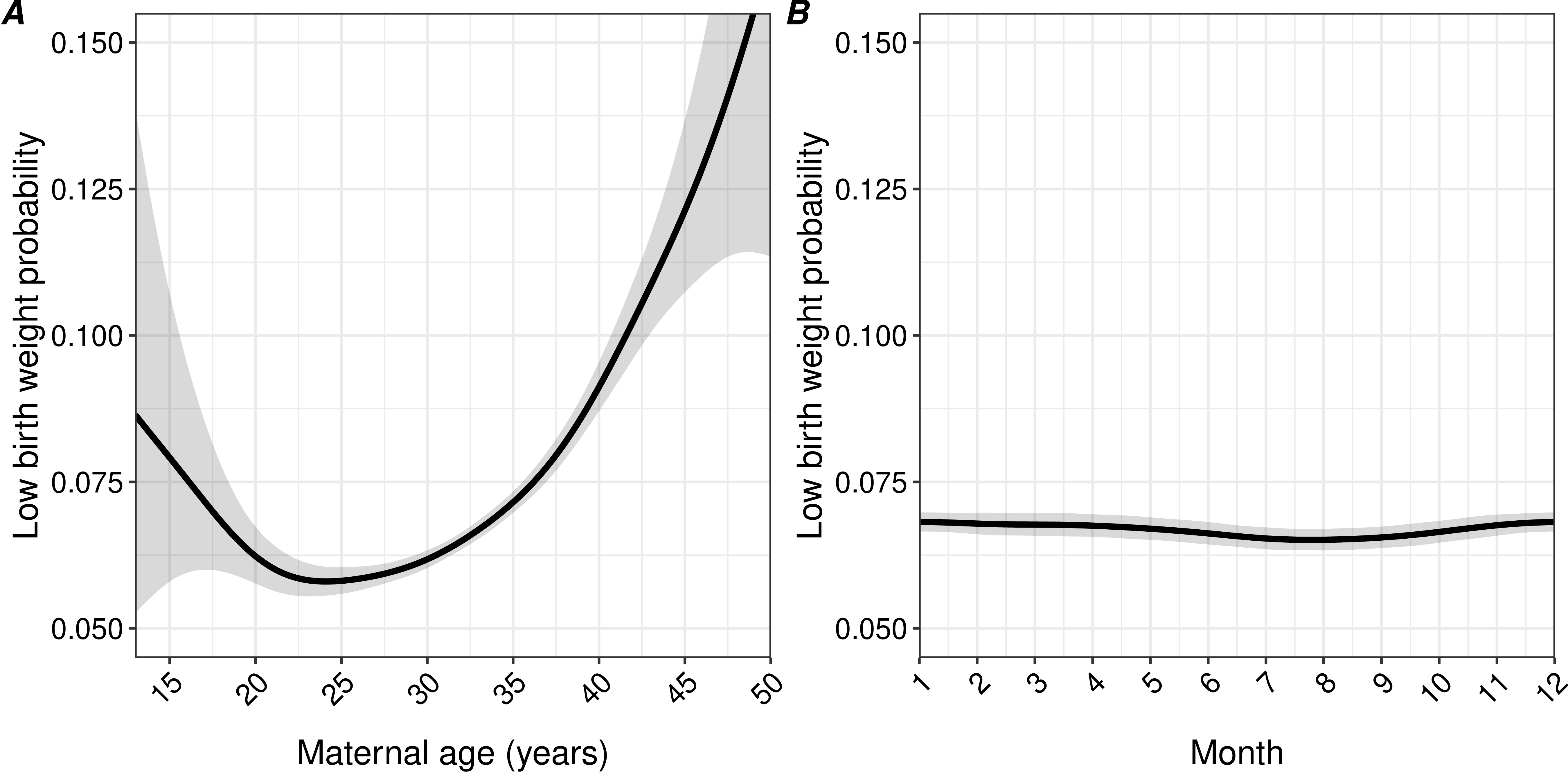


**A**: maternal age. **B**: Seasonality.

**Table S10:** trimester effect of crises on low birth weight.

|  |  |  | **95% CI** | |  |
| --- | --- | --- | --- | --- | --- |
| **Crisis** | **Trimester** | **OR** | **lci** | **uci** | **d** |
| **Heatwave** | **First** | 0.97 | 0.92 | 1.02 | 0.02 |
| **Great Recession** |  | 1.00 | 0.94 | 1.05 | 0.00 |
| **COVID-19** |  | 1.05 | 0.99 | 1.12 | 0.03 |
| **Heatwave** | **Last** | 1.00 | 0.95 | 1.05 | 0.00 |
| **Flu** |  | 0.98 | 0.92 | 1.04 | 0.01 |
| **COVID-19** |  | 0.94 | 0.88 | 1.00 | 0.04 |

OR: Odds-ratio, lci: lower confidence interval, uci: upper confidence interval,

d: Cohen’s d. Corresponding models are model 4.1.A and 4.1.B. Cohen’s d >0.1, >0.3,

>0.5 are respectively considered small, moderate and large effect sizes.

**Sensitivity analysis on first parities only:**

**Table S11**: Birth weight linear regression GAM among first parities only (model 1.3).

|  |  |  | **95% CI (g)** | |  |
| --- | --- | --- | --- | --- | --- |
| **Variable** | **Category** | **beta** | **lci** | **uci** | **d** |
| Time (by month) |  | 0.04 | 0.01 | 0.08 | 0.00 |
| SSEP (/10 points) |  | 7.78 | 6.13 | 9.43 | 0.01 |
| Altitude (/100m) |  | -7.24 | -7.97 | -6.50 | 0.02 |
| Sex (ref: male) | Female | -123.30 | -125.81 | -120.78 | 0.12 |
| Urban (ref: rural) | Urban | 7.14 | 4.50 | 9.78 | 0.01 |
| Language region (ref: German) | French | -45.00 | -48.07 | -41.93 | 0.04 |
|  | Italian | -72.08 | -79.04 | -65.13 | 0.03 |
| Maternal nationality (ref: Swiss) | Africa | 25.84 | 17.58 | 34.09 | 0.01 |
|  | Asia | -32.78 | -39.60 | -25.96 | 0.01 |
|  | Europe | 43.51 | 40.69 | 46.33 | 0.04 |
|  | Northern America | 86.36 | 70.79 | 101.93 | 0.01 |
|  | Southern/Central America | 30.91 | 21.84 | 39.97 | 0.01 |
| Civil status (ref: married) | Single | -37.16 | -39.97 | -34.35 | 0.03 |
| Heatwave (ref: 0) | 1 | 9.78 | 5.45 | 14.11 | 0.01 |
| Great Recession (continuous) |  | -11.63 | -24.14 | 0.88 | 0.00 |
| COVID (continuous) |  | 16.26 | 7.21 | 25.31 | 0.00 |
| **Smooth variables** |  |  |  |  | **p-value** |
| Maternal age (years) |  |  |  |  | <0.0001 |
| Seasonality (month) |  |  |  |  | 0.05 |
| *n*=622’748 | | | | | |

95%CI: 95% Confidence interal, lci: lower confidence interval, uci: upper confidence interval, d: Cohen’s d.

Cohen’s d >0.1, >0.3, >0.5 are respectively considered small, moderate and large effect sizes. Great

Recession and COVID-19 exposure variables are relative to pregnancy duration (values between 0 and 1).

SSEP scale goes from 23.6 to 86.7, by 10 points increase.

**Figure S13**: Birth weight linear regression GAM among first parities only (model 1.3), smooth variables.


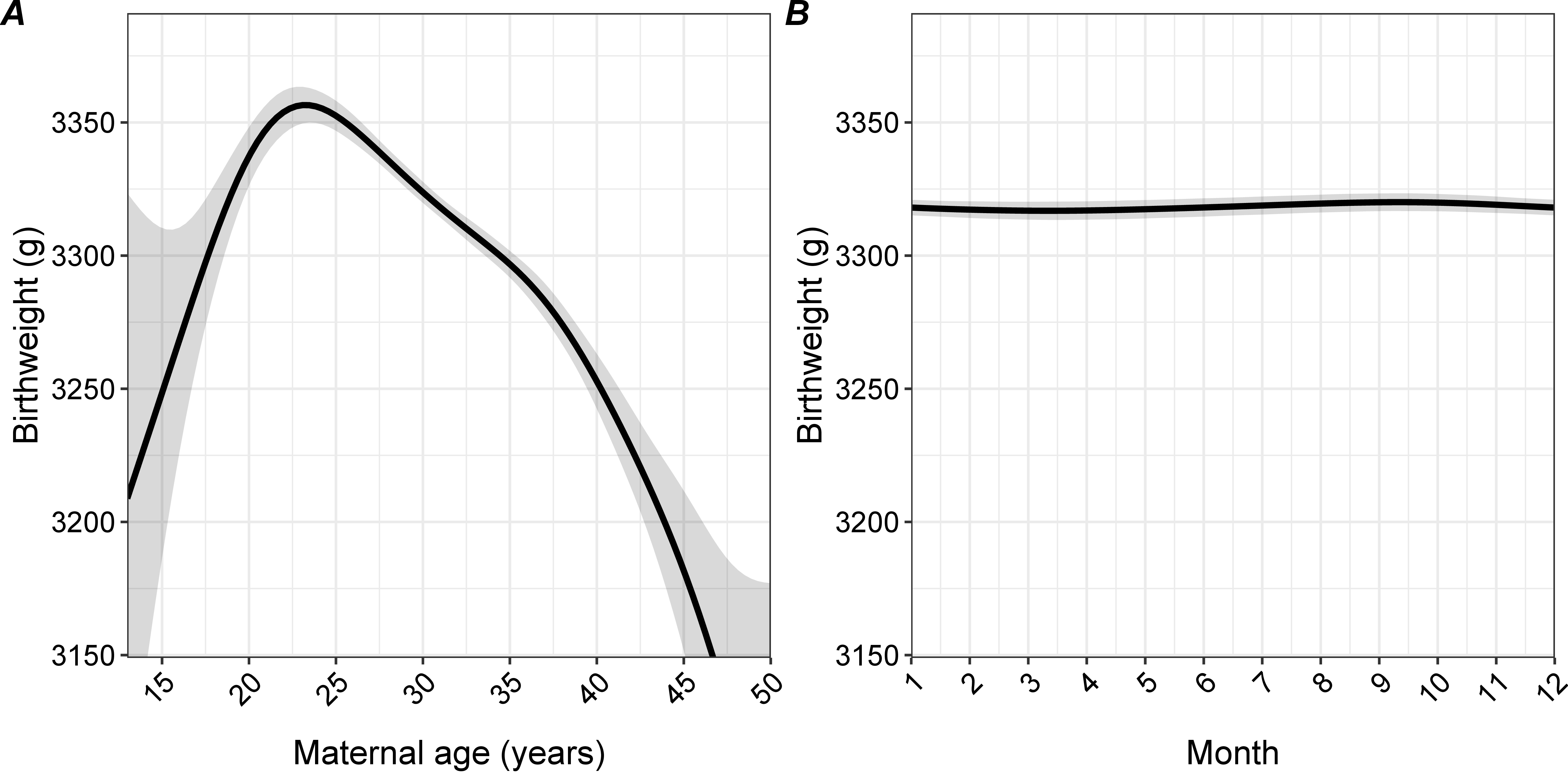


**A**: maternal age. **B:** Seasonality**.**

**Table S12:** Preterm birth logistic regression GAM among first parities only (model 2.3).

|  |  |  | **95% CI** | |  |
| --- | --- | --- | --- | --- | --- |
| **Variable** | **Category** | **OR** | **lci** | **uci** | **d** |
| Time (by month) |  | 1.00 | 1.00 | 1.00 | 0.00 |
| SSEP (/10 points) |  | 0.95 | 0.94 | 0.97 | 0.03 |
| Altitude (/100m) |  | 1.00 | 0.99 | 1.01 | 0.00 |
| Sex (ref: male) | Female | 0.83 | 0.82 | 0.85 | 0.10 |
| Urban (ref: rural) | Urban | 0.92 | 0.90 | 0.94 | 0.05 |
| Language region (ref: German) | French | 0.98 | 0.96 | 1.01 | 0.01 |
|  | Italian | 0.94 | 0.89 | 1.00 | 0.03 |
| Maternal nationality (ref: Swiss) | Africa | 0.93 | 0.87 | 1.00 | 0.04 |
|  | Asia | 0.93 | 0.87 | 0.98 | 0.04 |
|  | Europe | 0.89 | 0.87 | 0.92 | 0.06 |
|  | Northern America | 0.79 | 0.69 | 0.91 | 0.13 |
|  | Southern/Central America | 0.96 | 0.89 | 1.04 | 0.02 |
| Civil status (ref: married) | Single | 1.11 | 1.09 | 1.14 | 0.06 |
| Heatwave (ref: 0) | 1 | 0.85 | 0.82 | 0.88 | 0.09 |
| Great Recession (continuous) |  | 1.02 | 0.92 | 1.12 | 0.01 |
| COVID (continuous) |  | 0.98 | 0.91 | 1.05 | 0.01 |
| **Smooth variables** |  |  |  |  | **p-value** |
| Maternal age (years) |  |  |  |  | <0.0001 |
| Seasonality (month) |  |  |  |  | <0.0001 |
| *n*=622’748 | | | | | |

95%CI: 95% Confidence interal, lci: lower confidence interval, uci: upper confidence interval,

d: Cohen’s d. Cohen’s d >0.1, >0.3, >0.5 are respectively considered small, moderate and

large effect sizes. Great Recession and COVID-19 exposure variables are relative to pregnancy

duration (values between 0 and 1). SSEP scale goes from 23.6 to 86.7, by 10 points increase.

**Figure S14:** Preterm birth logistic regression GAM among first parities only (model 2.3).


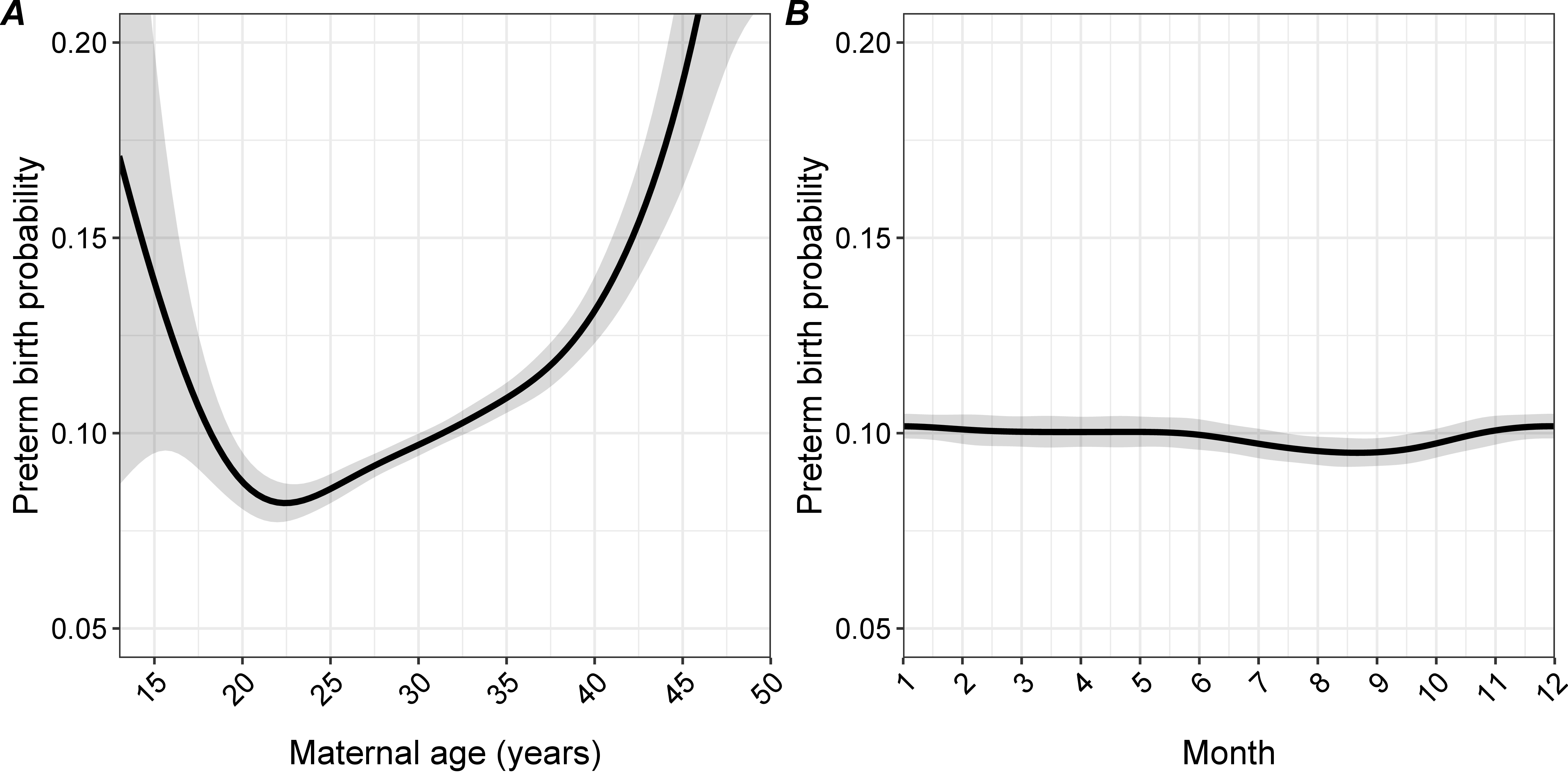


**A**: maternal age. **B:** Seasonality**.**

**Table S13:** trimester effect of crises on birth weight and preterm birth among first parities only.

|  |  | **Birth weight** | | | | **Preterm birth** | | | |
| --- | --- | --- | --- | --- | --- | --- | --- | --- | --- |
| **Crisis** | **Trimester** | **beta (g)** | **lci (g)** | **uci (g)** | **d** | **OR** | **lci** | **uci** | **d** |
| **Heatwave** | **First** | -1.17 | -8.66 | 6.33 | 0.00 | 0.96 | 0.90 | 1.02 | 0.02 |
| **Great Recession** |  | 4.60 | -3.67 | 12.87 | 0.00 | 0.95 | 0.87 | 1.03 | 0.03 |
| **COVID-19** |  | -0.69 | -9.31 | 7.93 | 0.00 | 1.06 | 0.99 | 1.14 | 0.03 |
| **Heatwave** | **Last** | -11.65 | -18.83 | -4.47 | 0.00 | 1.04 | 0.97 | 1.10 | 0.02 |
| **Great Recession** |  | -7.09 | -15.72 | 1.54 | 0.00 | 0.96 | 0.88 | 1.04 | 0.02 |
| **COVID-19** |  | 19.47 | 10.53 | 28.41 | 0.01 | 0.99 | 0.92 | 1.06 | 0.01 |

OR: Odds-ratio, lci: lower confidence interval, uci: upper confidence interval, d: Cohen’s

d. Corresponding models are model 1.3.A and 1.3.B (birth weight), and model 2.3.A and

2.3.B (preterm birth). Cohen’s d >0.1, >0.3, >0.5 are respectively considered small,

moderate and large effect sizes.

**Table S14**: summary of all models used

| name | description | outcome | crises variables | data |
| --- | --- | --- | --- | --- |
| 1.1 | main model | birth weight | heatwave, Great Recession, COVID * | livebirths |
| 1.1.A | first trimester crisis exposure |  | heatwave (1st trim), Great Recession (1st trim), COVID (1st trim) * |  |
| 1.1.B | last trimester crisis exposure |  | heatwave (last trim), Great Recession (last trim), COVID (last trim) * |  |
| 1.2 | flu instead of great recession |  | heatwave, Flu, COVID * |  |
| 1.2.A | first trimester exposure (flu instead of great recession) |  | heatwave (1^st^ trim), Flu (1^st^ trim), COVID (1^st^ trim) * |  |
| 1.2.B | last trimester exposure (flu instead of great recession) |  | heatwave (last trim), Flu (last trim), COVID (last trim) * |  |
| 1.3 | sensitivity analysis : primiparous women |  | heatwave, Great Recession, COVID ** | livebirths & primiparous women |
| 1.3.A | first trimester exposure (primiparous women) |  | heatwave (1st trim), Great Recession (1st trim), COVID (1st trim) ** |  |
| 1.3.B | last trimester exposure (primiparous women) |  | heatwave (last trim), Great Recession (last trim), COVID (last trim) ** |  |
| 2.1 | main model | preterm birth | heatwave, Great Recession, COVID * | livebirths |
| 2.1.A | first trimester exposure |  | heatwave (1st trim), Great Recession (1st trim), COVID (1st trim) * |  |
| 2.1.B | last trimester exposure |  | heatwave (last trim), Great Recession (last trim), COVID (last trim) * |  |
| 2.2 | flu instead of great recession |  | heatwave, Flu, COVID * |  |
| 2.2.A | first trimester exposure (flu instead of great recession) |  | heatwave (1^st^ trim), Flu (1^st^ trim), COVID (1^st^ trim) * |  |
| 2.2.B | last trimester exposure (flu instead of great recession) |  | heatwave (last trim), Flu (last trim), COVID (last trim) * |  |
| 2.3 | sensitivity analysis : primiparous women |  | heatwave, Great Recession, COVID ** | livebirths & primiparous women |
| 2.3.A | first trimester exposure (primiparous women) |  | heatwave (1st trim), Great Recession (1st trim), COVID (1st trim) ** |  |
| 2.3.B | last trimester exposure (primiparous women) |  | heatwave (last trim), Great Recession (last trim), COVID (last trim) ** |  |
| 3.1 | main model | stillbirth | heatwave, Great Recession, COVID ** | all births |
| 3.1.A | first trimester crisis exposure |  | heatwave (1st trim), Great Recession (1st trim), COVID (1st trim) ** |  |
| 3.1.B | last trimester crisis exposure |  | heatwave (last trim), Great Recession (last trim), COVID (last trim) ** |  |
| 3.2 | flu instead of great recession |  | heatwave, Flu, COVID ** |  |
| 3.2.A | first trimester exposure (flu instead of great recession) |  | heatwave (1^st^ trim), Flu (1^st^ trim), COVID (1^st^ trim) ** |  |
| 3.2.B | last trimester exposure (flu instead of great recession) |  | heatwave (last trim), Flu (last trim), COVID (last trim) ** |  |
| 4.1 | Model with birth weight as a binary outcome (<2’500g) | Low birth weight | heatwave, Great Recession, COVID * | livebirths |
| 4.1.A | first trimester crisis exposure |  | heatwave (1st trim), Great Recession (1st trim), COVID (1st trim) * |  |
| 4.1.B | last trimester crisis exposure |  | heatwave (1st trim), Great Recession (1st trim), COVID (1st trim) * |  |

*: models were adjusted for time, SSEP, altitude, parity, sex, urbanity, language region, maternal nationality, civil status, maternal age, seasonality. **: models were adjusted for time, SSEP, altitude, sex, urbanity, language region, maternal nationality, civil status, maternal age, seasonality.

**Table S15**: **share of each gestational age category in the analysed population**

| **Birthyear** |  | **2007** | **2008** | **2009** | **2010** | **2011** | **2012** | **2013** | **2014** | **2015** | **2016** | **2017** | **2018** | **2019** | **2020** | **2021** | **2022** |
| --- | --- | --- | --- | --- | --- | --- | --- | --- | --- | --- | --- | --- | --- | --- | --- | --- | --- |
|  | **Gestational age (weeks)** | | | | | | | | | | | | | | | | |
| **37-38** |  | 7.8 | 7.8 | 7.4 | 6.7 | 6.6 | 6.8 | 6.6 | 6.6 | 6.3 | 6.7 | 6.5 | 6.5 | 6.4 | 6.4 | 6.5 | 6.1 |
| **38-39** |  | 22.0 | 22.7 | 21.6 | 21.2 | 20.7 | 21.2 | 20.8 | 20.9 | 20.9 | 21.2 | 20.5 | 20.4 | 20.3 | 20.7 | 20.2 | 20.3 |
| **39-40** |  | 26.9 | 27.4 | 26.9 | 28.1 | 28.3 | 28.2 | 27.7 | 27.7 | 28.1 | 27.7 | 27.7 | 27.9 | 27.7 | 27.9 | 28.6 | 28.4 |
| **40-41** |  | 29.0 | 28.4 | 29.3 | 29.3 | 29.6 | 29.4 | 29.8 | 29.6 | 29.4 | 29.2 | 29.9 | 29.9 | 30.0 | 29.8 | 29.5 | 29.6 |
| **>=41** |  | 14.4 | 13.7 | 14.8 | 14.7 | 14.8 | 14.3 | 15.0 | 15.3 | 15.4 | 15.2 | 15.4 | 15.3 | 15.5 | 15.3 | 15.2 | 15.5 |

This dataset excludes stillbirths and preterm births. The share is in %. *n*=1’202’936
